# Artificial intelligence for classification of temporal lobe epilepsy with ROI-level MRI data: A worldwide ENIGMA-Epilepsy study

**DOI:** 10.1016/j.nicl.2021.102765

**Published:** 2021-07-24

**Authors:** Ezequiel Gleichgerrcht, Brent C. Munsell, Saud Alhusaini, Marina K.M. Alvim, Núria Bargalló, Benjamin Bender, Andrea Bernasconi, Neda Bernasconi, Boris Bernhardt, Karen Blackmon, Maria Eugenia Caligiuri, Fernando Cendes, Luis Concha, Patricia M. Desmond, Orrin Devinsky, Colin P. Doherty, Martin Domin, John S. Duncan, Niels K. Focke, Antonio Gambardella, Bo Gong, Renzo Guerrini, Sean N. Hatton, Reetta Kälviäinen, Simon S. Keller, Peter Kochunov, Raviteja Kotikalapudi, Barbara A.K. Kreilkamp, Angelo Labate, Soenke Langner, Sara Larivière, Matteo Lenge, Elaine Lui, Pascal Martin, Mario Mascalchi, Stefano Meletti, Terence J. O'Brien, Heath R. Pardoe, Jose C. Pariente, Jun Xian Rao, Mark P. Richardson, Raúl Rodríguez-Cruces, Theodor Rüber, Ben Sinclair, Hamid Soltanian-Zadeh, Dan J. Stein, Pasquale Striano, Peter N. Taylor, Rhys H. Thomas, Anna Elisabetta Vaudano, Lucy Vivash, Felix von Podewills, Sjoerd B. Vos, Bernd Weber, Yi Yao, Clarissa Lin Yasuda, Junsong Zhang, Paul M. Thompson, Sanjay M. Sisodiya, Carrie R. McDonald, Leonardo Bonilha, ENIGMA-Epilepsy Working Group, Andre Altmann, Chantal Depondt, Marian Galovic, Sophia I. Thomopoulos, Roland Wiest

**Affiliations:** a Centre for Medical Image Computing, Department of Medical Physics and Biomedical Engineering, University College London, London, UK; b Department of Neurology, Hôpital Erasme, Université Libre de Bruxelles, Brussels, Belgium; c Department of Clinical and Experimental Epilepsy, UCL Queen Square Institute of Neurology, London, UK; d Department of Neurology, University Hospital Zurich, Zurich, Switzerland; e Imaging Genetics Center, Mark and Mary Stevens Institute for Neuroimaging and Informatics, Keck School of Medicine, University of Southern California, Marina del Rey, CA, USA; f Department of Neurology and Epileptology, Hertie Institute for Clinical Brain Research, University of Tübingen, Tübingen, Germany; a Department of Neurology, Medical University of South Carolina, Charleston, SC, USA; b Department of Psychiatry, University of North Carolina at Chapel Hill, NC, USA; c Department of Computer Science, University of North Carolina at Chapel Hill, NC, USA; d Neurology Department, Yale University School of Medicine, New Haven, CT, USA; e Department of Molecular and Cellular Therapeutics, The Royal College of Surgeons in Ireland, Dublin, Ireland; f Department of Neurology and Neuroimaging Laboratory, University of Campinas - UNICAMP, Campinas, SP, Brazil; g Magnetic Resonance Image Core Facility, Institut d’Investigacions Biomèdiques August Pi i Sunyer (IDIBAPS), Universitat de Barcelona, Barcelona, Spain; h Department of Radiology of Center of Image Diagnosis (CDIC), Hospital Clinic de Barcelona, Barcelona, Spain; i Department of Diagnostic and Interventional Neuroradiology, University Hospital Tübingen, Tübingen, Germany; j Neuroimaging of Epilepsy Laboratory, Montreal Neurological Institute, McGill University, Montreal, QC, Canada; k McConnell Brain Imaging Center, Montreal Neurological Institute, McGill University, Montreal, QC, Canada; l Psychiatry and Psychology, Mayo Clinic, Jacksonville, FL, USA; m Neuroscience Research Center, Department of Medical and Surgical Sciences, University “Magna Græcia“ of Catanzaro, Catanzaro, Italy; n Instituto de Neurobiología, Universidad Nacional Autónoma de México, Querétaro, Mexico; o Department of Radiology, Royal Melbourne Hospital, University of Melbourne, Melbourne, VIC, Australia; p Department of Neurology, Langone School of Medicine, New York University, New York, NY, USA; q Trinity College Dublin, School of Medicine, Dublin, Ireland; r FutureNeuro SFI Research Centre for Rare and Chronic Neurological Diseases, Dublin, Ireland; s Functional Imaging Unit, Department of Diagnostic Radiology and Neuroradiology, University Medicine Greifswald, Greifswald, Germany; t Department of Clinical and Experimental Epilepsy, UCL Queen Square Institute of Neurology, London, UK; u University Medicine Göttingen, Clinical Neurophysiology, Göttingen, Germany; v Institute of Neurology, University “Magna Græcia“ of Catanzaro, Catanzaro, Italy; w Department of Radiology, BC Children’s Hospital, University of British Columbia, Vancouver, BC, Canada; x Neuroscience Department, University of Florence, Florence, Italy; y Center for Multimodal Imaging and Genetics, University of California, San Diego, La Jolla, CA, USA; z Kuopio University Hospital, Member of EpiCARE ERN, Kuopio, Finland; aa Institute of Clinical Medicine, Neurology, University of Eastern Finland, Kuopio, Finland; ab Institute of Systems, Molecular and Integrative Biology, University of Liverpool, Liverpool, UK; ac The Walton Centre NHS Foundation Trust, Liverpool, UK; ad Department of Psychiatry, University of Maryland School of Medicine, Baltimore, MD, USA; ae Department of Clinical Neurophysiology, University Hospital Göttingen, Goettingen, Germany; af Department of Neurology and Epileptology, Hertie Institute for Clinical Brain Research, University Hospital Tübingen, Tübingen, Germany; ag Institute for Diagnostic Radiology and Neuroradiology, University Medicine Greifswald, Greifswald, Germany; ah Institute for Diagnostic and Interventional Radiology, Pediatric and Neuroradiology, University Medical Centre Rostock, Rostock, Germany; ai Pediatric Neurology, Neurogenetics and Neurobiology Unit and Laboratories, Children’s Hospital A. Meyer-University of Florence, Florence, Italy; aj Functional and Epilepsy Neurosurgery Unit, Neurosurgery Department, Children’s Hospital A. Meyer-University of Florence, Florence, Italy; ak ‘Mario Serio’ Department of Clinical and Experimental Medica Sciences, University of Florence, Florence, Italy; al Department of Biomedical, Metabolic, and Neural Sciences, University of Modena and Reggio Emilia, Modena, Italy; am Neurology Unit, OCB Hospital, AOU Modena, Modena, Italy; an Department of Neuroscience, Monash University, Melbourne, VIC, Australia; ao The Department of Medicine (The Royal Melbourne Hospital), The University of Melbourne, Parkville, VIC, Australia; ap Department of Neurology, Alfred Health, Melbourne, VIC, Australia; aq Department of Psychiatry, University of California San Diego, La Jolla, CA, USA; ar Division of Neuroscience, King’s College London, London, UK; as Montreal Neurological Institute and Hospital, McGill University, Montreal, QC, Canada; at Department of Epileptology, University Hospital Bonn, Bonn, Germany; au Radiology and Research Administration, Henry Ford Health System, Detroit, MI, USA; av School of Electrical and Computer Engineering, College of Engineering, University of Tehran, Tehran, Iran; aw SA MRC Unit on Risk & Resilience in Mental Disorders, Department of Psychiatry & Neuroscience Institute, University of Cape Town, Cape Town, South Africa; ax IRCCS Istituto ‘G. Gaslini’, Genova, Italy; ay Department of Neurosciences, Rehabilitation, Ophthalmology, Genetics, Maternal and Child Health, University of Genova, Genova, Italy; az School of Computing, Newcastle University, Newcastle Upon Tyne, UK; ba Institute of Translational and Clinical Research, Newcastle University, Newcastle Upon Tyne, UK; bb Department of Neurology, Epilepsy Center, University Medicine Greifswald, Greifswald, Germany; bc Centre for Medical Image Computing, Department of Computer Science, University College London, London, UK; bd Neuroradiological Academic Unit, UCL Queen Square Institute of Neurology, University College London, London, UK; be Institute of Experimental Epileptology and Cognition Research, University of Bonn, Bonn, Germany; bf Cognitive Science Department, School of Informatics, Xiamen University, Xiamen, China; bg Imaging Genetics Center, Mark and Mary Stevens Institute for Neuroimaging and Informatics, Keck School of Medicine, University of Southern California, Marina del Rey, CA, USA; bh UCL Queen Square Institute of Neurology, London, UK; bi Chalfont Centre for Epilepsy, Bucks, UK

**Keywords:** Epilepsy, Temporal lobe epilepsy, Machine learning, Artificial inteligence

## Abstract

Artificial intelligence has recently gained popularity across different medical fields to aid in the detection of diseases based on pathology samples or medical imaging findings. Brain magnetic resonance imaging (MRI) is a key assessment tool for patients with temporal lobe epilepsy (TLE). The role of machine learning and artificial intelligence to increase detection of brain abnormalities in TLE remains inconclusive. We used support vector machine (SV) and deep learning (DL) models based on region of interest (ROI-based) structural (n = 336) and diffusion (n = 863) brain MRI data from patients with TLE with (“lesional”) and without (“non-lesional”) radiographic features suggestive of underlying hippocampal sclerosis from the multinational (multi-center) ENIGMA-Epilepsy consortium. Our data showed that models to identify TLE performed better or similar (68–75%) compared to models to lateralize the side of TLE (56–73%, except structural-based) based on diffusion data with the opposite pattern seen for structural data (67–75% to diagnose vs. 83% to lateralize). In other aspects, structural and diffusion-based models showed similar classification accuracies. Our classification models for patients with hippocampal sclerosis were more accurate (68–76%) than models that stratified non-lesional patients (53–62%). Overall, SV and DL models performed similarly with several instances in which SV mildly outperformed DL. We discuss the relative performance of these models with ROI-level data and the implications for future applications of machine learning and artificial intelligence in epilepsy care.

## Highlights

• Machine learning and artificial intelligence have gained popularity for medical applications.
• We applied support vector machine (SV) and deep learning (DL) in termporal lobe epilepsy (TLE)
• Structural and diffusion-based models showed similar classification accuracies.
• Diffusion-based models to diagnose TLE performed better or similar compared to models to lateralize TLE.
• Models for patients with hippocampal sclerosis were more accurate than models that stratified non-lesional patients.

## 1 Introduction

Temporal lobe epilepsy (TLE) is the most common focal onset epilepsy in adults (Tellez-Zenteno and Hernandez-Ronquillo, 2012). Although hippocampal sclerosis is a common pathological finding in TLE (Babb and Brown, 1987, Blumcke, I.,et.al., 2017), structural abnormalities in TLE are not restricted to the medial temporal region and may extend to the neocortex (Blanc et al., 2011, Margerison and Corsellis, 1966). Magnetic resonance imaging (MRI) provides a non-invasive approach to study epilepsy-related pathology, and quantitative analyses of MRI data have shown widespread grey and white matter abnormalities beyond the hippocampal structure (Whelan et al., 2018). Tissue volume abnormalities occur both proximal and distal to medial temporal lobe circuits with histopathologically confirmed hippocampal sclerosis and EEG confirmation of concordant seizure laterality (Ahmadi et al., 2009, Bernhardt et al., 2010, Bernhardt et al., 2008, Bonilha et al., 2010a, Bonilha et al., 2010b, Bonilha et al., 2003, Caligiuri et al., 2016, Focke et al., 2008, Lin et al., 2007, McDonald et al., 2008b).

Extra-hippocampal network structural abnormalities are associated with cognitive deficits (McDonald et al., 2008a, Rodriguez-Cruces et al., 2020, Yogarajah et al., 2008) as well as treatment response phenotypes (Bernhardt et al., 2015, Bonilha et al., 2015, Bonilha and Keller, 2015, Gleichgerrcht et al., 2018, Munsell et al., 2015). Methods that can accurately detect, quantify, and profile structural abnormalities at an individual level can be relevant for multiple aspects of translational epilepsy research and may help guide clinical management in the future (Gleichgerrcht and Bonilha, 2017, Gleichgerrcht et al., 2015).

Artificial intelligence offers a promising approach to assess subtle abnormalities in neuroimaging data. In particular, machine learning and deep learning may enable the detection of patterns in an automated way that would otherwise be difficult to discern through qualitative evaluations. Moreover, training and evaluation of these models generally follow a framework that emphasizes out-of-sample prediction performance, where a trained model is tested in an independent dataset to determine whether conclusions from one sample may be generalizable and not due to biases from a small sampled population. Artificial intelligence has gained significant popularity in medicine as it can achieve detection accuracies similar to, or sometimes better than, human experts in the diagnosis of many diseases, such as lung cancer (Coudray et al., 2018), skin cancer (Haenssle et al., 2018), and retinal abnormalities (Gulshan et al., 2016). More recently, applications of artificial intelligence and machine learning have expanded in the field of epilepsy (Abbasi and Goldenholz, 2019, Focke et al., 2012, Gleichgerrcht et al., 2020, Gleichgerrcht et al., 2018, Rudie et al., 2015).

Can machine learning and deep learning accurately classify individual brain images into a pattern typical of TLE, or even lateralize TLE? This question remains unanswered, partly due to the smaller sample size of TLE datasets compared with other diseases (e.g., pneumonia, skin cancer, retinal abnormalities). The present study uses a novel initiative, the *Enhancing NeuroImaging Genetics through Meta-Analysis Epilepsy project* (ENIGMA-Epilepsy). ENIGMA-Epilepsy is a global initiative, which pools individually collected samples from sites across the world with uniform image processing and phenotypic characterizations (Thompson et al., 2020). ENIGMA-Epilepsy offers the unique advantage of sample size: it constituted the first collection of quantitative imaging data in common epilepsy syndromes, including persons with TLE, with more than one thousand data points derived from neuroimaging studies of patients as well as healthy controls with structural and/or diffusion imaging available, together with basic clinical and socio-demographic phenotypes (Hatton et al., 2020, Lariviere et al., 2020, Whelan et al., 2018). However, in its current form, it has the inherent limitation of containing only region of interest (ROI)-based data, i.e. data collected at the level of a “brain region” as defined by an atlas with predefined anatomical boundaries. This is an important trade-off: the large sample size compensates for the lower resolution of the data (i.e., data summarized at the *regional* level), although efforts to obtain raw image-based analysis are underway.

In this study, we investigated if artificial intelligence applied to ENIGMA ROI-wise data could be used to 1) classify individuals as either having TLE or being healthy controls and 2) lateralize into right versus left TLE. These are important steps in the assessment of persons with epilepsy as this can have consequences not only for their medical but also their potential surgical management if they were to remain refractory to anti-seizure medications. Contrary to prior studies ENIGMA-epilepsy which focused on ROI-level discrimination of groups, this study sought to predict group classification based on multivariate machine learning approaches. To achieve these goals, we used morphological features from T1-weighted data as well as diffusion MRI parameters sensitive to microstructural variations. We hypothesized that ML could be a viable tool for TLE classification and lateralization. We further extended our investigation by comparing the classification accuracy of different ML methods. If the hypothesis was to be confirmed, the study could serve as a guide for future multi-center large cohort initiatives.

## 2 Materials and methods

### 2.1 Participant inclusion

This retrospective study included data from adult TLE patients (aged from 18 to 70), identified using the International League Against Epilepsy classification criteria (Berg et al., 2010). Diagnoses were based on the standard of care assessment batteries at each site; this included neurophysiology and neuroimaging studies as needed on an individual basis. Based on these data, each patient was clinically classified a priori by the medical team at each site into one of four groups: (i) left-sided TLE with MRI signs of HS (TLE-HS-L), (ii) right-sided TLE with HS (TLE-HS-R), (iii) non-sclerosis left-sided TLE (TLE-NS-L), and (iv) non-sclerosis right-sided TLE (TLE-NS-R). Patients with TLE-NS were defined based on the absence of any abnormalities (including evidence of underlying hippocampal sclerosis) as deemed by each site based on conventional radiographic interpretation. Because structural MRI data collection for ENIGMA-Epilepsy initially classified patients in the TLE-NS group as “Other,” there were no structural data for TLE-NS as it is not possible to tease apart this subgroup from the remainder of the “Other” category members. Hence, only diffusion data are available for TLE-NS, as this label was implemented during the second wave of data collection. Patients with discordant imaging/neurophysiology data (e.g., left-sided onset seizures with neuroimaging findings suggestive of right-sided mesial temporal sclerosis) were not included except if histopathological confirmation allowed definitive classification. We excluded patients with progressive diseases (e.g., Rasmussen’s encephalitis), autoimmune etiology, malformations of cortical development, tumors, or previous neurosurgical intervention.

Healthy controls from each site had no prior history of psychiatric or neurological disorders. The Institutional Review Board (IRB) approval for pseudo-anonymized data collection and sharing was obtained individually at each site prior to enrollment into the consortium.

### 2.2 Structural imaging dataset

Data from 16 sites were included in the structural MRI analysis, totaling 336 patients with TLE (187 TLE-HS-L and 149 TLE-HS-R) and 631 research center-matched healthy controls. The locations, dates, and periods of participant recruitment as well as demographic and clinical characteristics of this sample are detailed in Whelan et al. (2018). The data analyzed for this study was derived from the pipeline implemented for the original structural MRI analysis of this cohort (Whelan et al., 2018) which essentially contemplated an analysis at each site using FreeSurfer 5.3.0, for automated analysis of brain structure (Fischl, 2012). Scanning details are provided in **Supplementary Table 1**. Using the Desikan-Killiany atlas, cortical thickness and surface area measures were extracted for 34 left-hemispheric grey matter (GM) regions of interest (ROI) and 34 right-hemispheric GM ROIs. In addition, subcortical volume measures were extracted for 8 left-hemispheric GM ROIs and 8 right-hemispheric GM ROIs (152 ROI features in total - i.e., 68 thickness measures, 68 surface area measures, and 16 subcortical volume measures). Visual inspections of cortical segmentations were conducted blinded to participants’ diagnosis following standardized ENIGMA protocols (available at http://enigma.ini.usc.edu) used in prior genetic studies of brain structure (Adams et al., 2016, Hibar et al., 2017, Hibar, D.P.,et.al., 2015, Stein, J.L., 2012) and large-scale case-control studies of neuropsychiatric illnesses and neurological disorders (Boedhoe, P.S.,et.al., 2017, Hoogman et al., 2017, Schmaal et al., 2017). Each site identified outliers by running a series of standardized bash scripts to detect participants with cortical thickness measures greater or less than 1.5 times the interquartile range. Outlier data were then visually inspected by overlaying the participant’s cortical segmentations on their whole-brain anatomical images. Structures were excluded if judged as inaccurately segmented by the local analyst. For this study, only participants with a full dataset (i.e., no omitted volumes) were analyzed.

### 2.3 Diffusion data set

Data from 21 sites were included in the diffusion imaging study, totaling 863 patients (320 TLE-HS-L, 268 TLE-HS-R, 162 TLE-NS-L, and 113 TLE-NS-R) and research center-matched healthy controls (1,069 with available fractional anisotropy (FA) data, 976 with available radial diffusivity (RD) data). The locations, dates, and periods of participant recruitment as well as demographic and clinical characteristics of this sample are reported in Hatton et al. (Hatton et al., 2020).

Scanner descriptions and acquisition protocols for all sites are provided in **Supplementary Table 2.** Each site conducted the preprocessing of diffusion-weighted images, including eddy current correction, echo-planar imaging (EPI)-induced distortion correction, and tensor estimation. Next, diffusion-tensor imaging (DTI) images were processed using the ENIGMA-DTI protocols. These image processing and quality control protocols are freely available at the ENIGMA-DTI^1^ and NITRC^2^ websites. For this study, we present fractional anisotropy (FA) and radial diffusivity (RD) metrics as these have recently shown the largest effect size differences across different epilepsy syndromes in a prior DTI mega-analysis by the ENIGMA-Epilepsy consortium (Hatton et al., 2020). Measures of FA and RD were hence derived for ROIs based on the Johns Hopkins University (JHU) atlas (Faria et al., 2010) which included left- and right-sided tracts as well as the midline structures of the body (BCC), genu (GCC), and splenium (SCC) of the corpus callosum (41 ROI features in total). For this study, only patients with a full dataset (i.e., no omitted volumes) were analyzed in each modality, although the exclusion of participants with at least one outlier value was modality-specific; that is, if a participant had an outlier FA value in a given ROI, that patient was still eligible for inclusion if the ROI value was a non-outlier in RD.

### 2.4 Multi-site data harmonization

The batch-effect correction tool “ComBat” was employed to harmonize between-site and between-protocol variations in structural and diffusion metrics, as previously done for other multi-site cohorts (Fortin et al., 2017, Hatton et al., 2020, Villalon-Reina et al., 2019, Zavaliangos-Petropulu et al., 2019). *ComBat* rescales the data for each scanner instance using a z-score transformation map common to all features using an empirical Bayes framework (Johnson et al., 2007) to adjust for variability across sites, assuming that all ROIs share the same common distribution. Prior to pipeline construction (Section 2.6), multi-site ROI, age, and sex differences were minimized using the ComBat technique.

### 2.5 Group imbalance correction

While the number of participants in both the structural and diffusion datasets was large, the number of healthy controls included was disproportionate relative to the number of patients with TLE, creating an imbalance problem that typically results in a classification model that is biased or overfitted to the samples in the majority group (i.e., HC in this study). To mitigate imbalance overfitting concerns, prior to pipeline construction (Section 2.6), we randomly selected ROI dataset samples (structural or diffusion) in the majority group to equal the number of minority (i.e., TLE) ROI dataset samples. This size-matching control cohort was randomly chosen from the larger pool of control participants at each of the 1000 iterations described below, maximizing selection by chance in each instance. We nonetheless acknowledge that there are alternatives approaches to prevent overfitting involving weighted penalties to the cost function of the classification algorithm based on the empirical class distribution which may achieve similar goals without the exclusion/inclusion of specific subsets of participants.

### 2.6 Configurable classification pipeline

**Supplementary figure 1:**
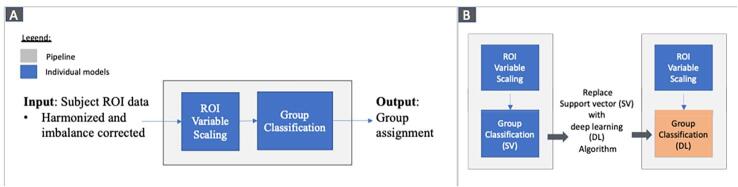


A configurable pipeline allows to exchange one model approach for another (e.g. support vector machine or deep learning) without creating ad hoc properties for each framework. In general, our pipeline sequentially applied two different models to identify structural or diffusion ROI group (e.g., HC vs. TLE-HS-L) differences at the subject level (Supplementary Figure 1). To apply our classification pipeline, a subject’s harmonized (Section 2.4) structural (Section 2.2) or diffusion (Section 2.3) ROI data was input into the pipeline, after which the data were sequentially processed by an ROI variable scaling model (Section 2.6.1) followed by a group classification model (Section 2.6.2), and the output was the subject’s assigned group (e.g., HC, TLE-HS-L, etc.).

To consistently evaluate classification pipeline performance, while mitigating biases related to software or computing differences, three strategies were taken: 1) a configurable pipeline approach (**Supplementary** Fig. 1B) was used that allowed individual models in the pipeline to be replaced with minimal coding changes, 2) the configurable pipeline was developed using well-known and well-tested publicly available Python software libraries (https://github.com/brent-munsell/enigma_roi), and 3) all the configurable pipeline results were generated on the same high-performance compute cluster (https://its.unc.edu/research-computing/longleaf-cluster/).

#### 2.6.1 ROI variable scaling

As the segmentation atlases employed ROIs of different volumes, and ROI measurements may be scaled differently (i.e. ROI volume measurements have larger values than ROI surface area or cortical thickness measurements) it was important to mitigate overfitting caused by scale differences. In other words, if the volume of ROI *A* is less than that of ROI *B*, the group classification model may be overfitted (or biased) to ROI *B* measurements solely due to size. To control for this, a simple minimum and maximum scaling technique was applied to each ROI: for each column (ROI) in the input matrix, 1) the minimum value across all participants was computed, 2) the minimum value was subtracted from each participant’s ROI value, 3) the maximum value was computed across all participants, and 4) each participant’s value was then divided by the maximum value.

#### 2.6.2 Group classification

**Supplementary figure 2:**
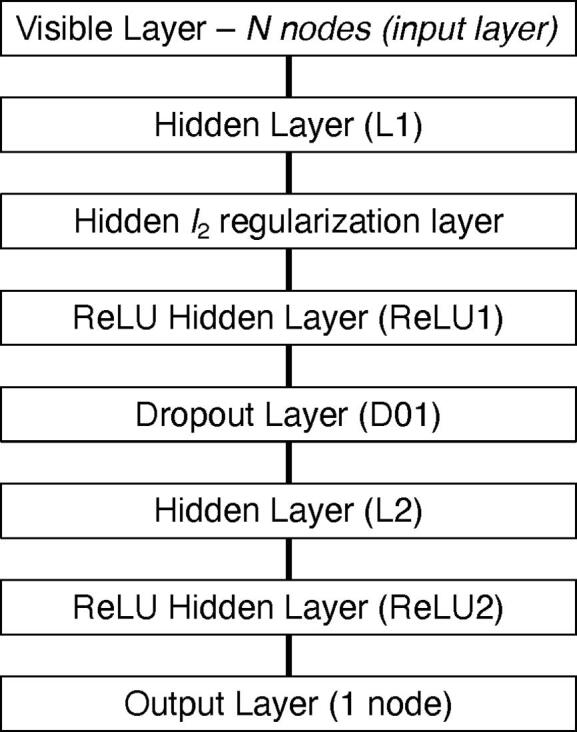


Two different ML algorithms were used in this study to classify individuals into one of two groups. Specifically, we employed two supervised ML techniques to perform group classification: a support vector classifier (SVC) and a deep-learning classifier (DLC). The SVC used a linear kernel and the optimal regularization parameter (C) was determined by a grid search procedure. In general, the DLC was a fully connected neural network that applied linear activation (Supplementary Figure 2) functions and featured: (i) one input layer where the number of nodes equaled the number of ROIs (152 for structural, 41 for diffusion), (ii) two hidden layers with the optimal number of nodes in each hidden layer determined by a grid search procedure (Section 2.6), and (iii) one output layer with one node representing healthy controls and one node representing one of the TLE patient groups (**Supplementary Note 1**). Notably, the optimal DLC learning and drop-out rates were also determined by a grid search.

#### 2.6.3 Grid search parameters

The following value ranges were used for grid search of optimal model parameters. For SVC, the C values went from 0.1 to 2.0 at 0.05 increments. For DLC, learning rate = 0.001 to 0.01 at 0.0005 increments; number of epochs = 100 to 800 at 50 increments; hidden layer 1 (L1) units = 5 to 100 at 5 increments; hidden layer 2 (L2) units = 3 to 20 at 1 increment; *l*_2_ regularization penalty = 0.1 to 0.7 at 0.1 increments; dropout rate = 0.2 to 0.5 at 0.1 increments.

### 2.7 Pipeline performance evaluation

**Fig. 1:**
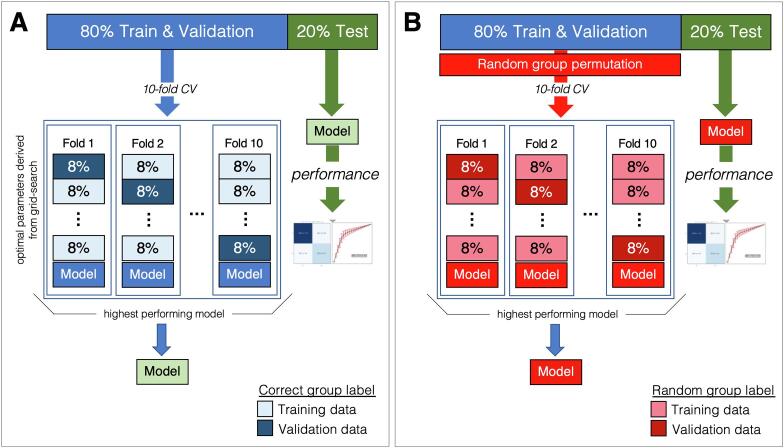
**Pipeline performance evaluation.** (**A**) Schematic illustration of a 10-fold grid search process to create an optimally trained pipeline with the highest classification accuracy; and (**B**) Schematic illustration of a 10-fold cross-validation process to create a pipeline using shuffled labels to yield a random distribution in order to assess statistical significance between models trained on real vs. permuted (random) data.

The performance of our pipeline was evaluated using guidelines recommended by Poldrack et al. (2019). In particular, a 10-fold cross-validation procedure that incorporated a grid search technique was used to evaluate pipeline classification performance (Fig. 1**A**). More specifically, given an M × N ComBat-harmonized and imbalance corrected *ROI data matrix,* where M (row) is the total number of participants and N (column) is the number of ROI variables, and an M × 1 *group label matrix* that holds the corresponding group label (e.g., HC = 0 and TLE-HS-L = 1) for each participant, the following steps were sequentially applied:

1) The rows of the data and group label matrices were randomly shuffled together (i.e., subject & group label maintained). A 80/20 percent stratified (based on group label) partition split was then applied to the matrices, where 80% were assigned to training and validation data, and 20% became test data. It is important to note that test data were not used to learn the ROI variable scaling parameters or the optimal group classification model parameters (found by the grid search outlined below).
2) Using only the training and validation data split, a 10-fold stratified grid search procedure was applied: one fold was selected as validation data and the remaining nine folds became the training data. An exhaustive grid search (i.e., iterations of all possible model parameter combinations to yield the most reliable set) was performed to estimate the optimal classification model parameters that yielded a pipeline with the highest classification accuracy. The model parameters included in the grid search were: number of nodes in hidden layers one and two, $l_{2}$ regularization penalty in hidden regularization layer, and random percent of nodes removed (i.e., dropped) in the hidden dropout layer (**Supplementary Note 1**)**.** This process was repeated until each fold had been selected as the validation fold, resulting in ten trained pipelines (i.e., one for each validation fold).
3) From the ten pipelines created by the grid search procedure above, the optimal pipeline was selected (i.e., one that had the highest classification accuracy) and the optimal model parameters identified (see **Supplementary Notes 1 through 4**).
4) For each subject in the test data matrix, the optimal pipeline was given the participant’s set of ROI variables and prompted to predict the participant’s group. To assess performance, a 2x2 confusion matrix was constructed using the *predicted* group label and *known* group label (i.e., the real diagnostic group) to calculate: positive predictive value (PPV), negative predictive value (NPV), sensitivity (SEN), specificity (SPC), area under the curve (AUC), and accuracy.

The four steps above were repeated 1,000 times to better estimate the stability of the optimal pipelines (including model parameters) found by the 10-fold cross-validation grid search.

### 2.8 Assessment of statistical significance

The performance of our pipeline was evaluated using a 10-fold cross-validation procedure that incorporated a *random* group label permutation to estimate the distribution of values under a null hypothesis (Fig. 1**B**). Given an M × N ComBat-harmonized and imbalance corrected *ROI data matrix* and an M × 1 *group label matrix*, the four steps above were carried out, where Steps 1 and 4 were identical to the ones previously outlined and Steps 2 and 3 differed as follows:

2) The training and validation group label matrix was randomly permuted, and then a 10-fold stratified cross-validation procedure was applied to our pipeline. In this cross-validation, a grid search was not performed. Instead, the highest performing model parameters for the correctly trained pipeline were used.
3) From the ten pipelines created by the cross-validation procedure, the non-optimal pipeline was selected (i.e., one that had the highest classification accuracy with random group label assignments).

As before, these four steps were repeated 1,000 times to better estimate the stability of the non-optimal pipelines found by the 10-fold cross-validation procedure. Using the test data set, the statistical significance of the difference in performance between the optimal and non-optimal pipelines was determined by evaluating how often the mean performance metric of the optimal pipeline was higher than the performance metric of the non-optimal pipeline. For instance, if the average classification accuracy of the optimal pipeline is greater than 98% of the classification accuracies obtained in the non-optimal pipeline, the probability that the optimal pipeline classification accuracy is due to chance is 2% or *p* = 0.02. The same analysis is applied to each performance metric (i.e., accuracy, PPV, NPV, etc.) in our study (Section 3.0).

In order to compare the performance of SVM against DL, we computed the DL accuracy and counted the number of SVM accuracies that were greater than the DL accuracy, then divided by the 1,000 iterations. For between-model comparisons, we report a frequency distribution comparison index (FDCI) where 0.5 demonstrates equipoise in the accuracies of the two approaches (i.e. SVM is as accurate as DL), whereas a FDCI value closer to 1 means that SV outperforms DL on almost every iteration, and vice versa**.**

### 2.9 ROI variable selection

For the SVC model, support vector coefficients were used to select the ROI variables that had the largest influence on group classification accuracy. For the DLC model, the backtracking technique (Hazlett et al., 2017, Munsell et al., 2020 Nov 1) was used to select the ROI variables that had the largest influence on group classification accuracy. Values were averaged across the 1000 iterations of model training**.**

## 3 Results

The results are organized by imaging modality (structural and diffusion, separately) and the specific group classification comparison that was performed. For each comparison, a 2 × 2 confusion matrix and a summary table displaying the six performance metrics, are shown as mean ± standard deviation (SD) calculated from the one-thousand 2 × 2 confusion matrices generated by the evaluation process (Section 2.6), and the one-thousand random 2 × 2 confusion matrices generated by our evaluation process that permutates the group labels (Section 2.6). For each performance metric, the statistical significance (p-value) is reported.

### 3.1 Structural data

**Fig. 2:**
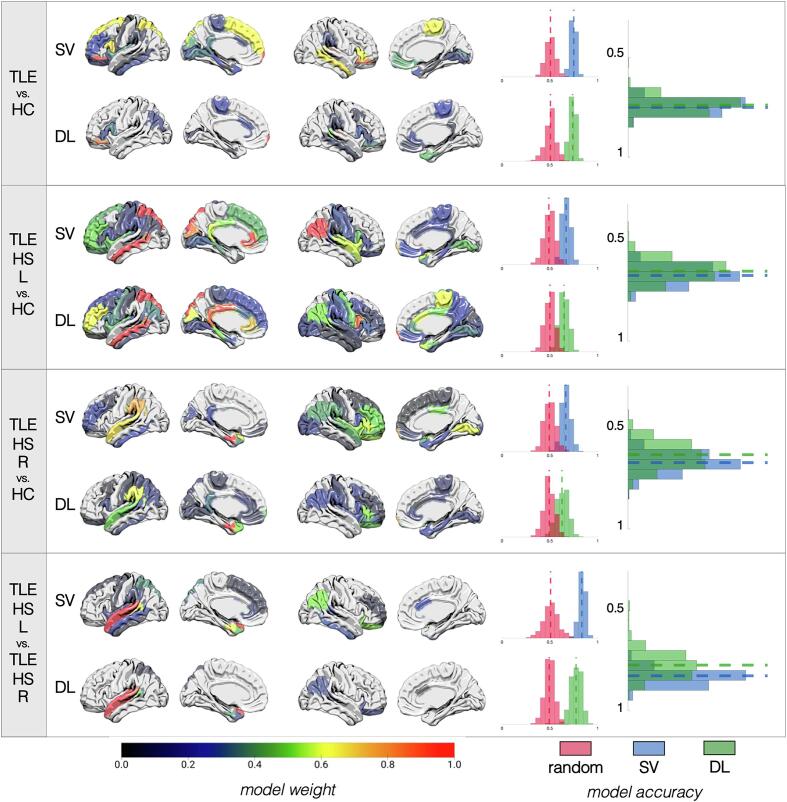
**Summary of results for models based on structural data.** For each of the four classifications (identified in the leftmost column), the top row shows the results for the support vector (SV) approach and the bottom row shows the results for the deep learning (DL) approach. Cortex projections are shown, from left to right, overlaid on a left lateral, left medial, right lateral, and right medial view, respectively. Projections correspond to model weights for each region of interest (ROI) based on the Desikan-Killiany atlas colored based on the colormap at the bottom of the figure and normalized from 0 to 1, where higher values (more red regions) correspond to the most influential ROIs for that particular model. For each classification approach, a distribution of accuracies is shown to the right for permutated labels (red) or real labels (blue for SV, green for DL). The rightmost graph compares the accuracy of SV vs. DL against each other. (For interpretation of the references to color in this figure legend, the reader is referred to the web version of this article.)

The performance of our classification pipelines based on structural data is summarized in Fig. 2 and **Supplementary Table 3** (optimal pipeline parameters available in **Supplementary Note 2**). SVM and DL pipelines demonstrated no significant differences in classification accuracy for TLE vs. HC comparisons. There was a 73 ± 2.9% (DL) to 75 ± 3.4% (SVM) accuracy to correctly distinguish all patients with TLE from HC based on GM volumes/thickness, although the accuracy was slightly lower (65–67%) when the model attempted to classify TLE patients based on the specific side of presumed seizure onset (i.e., TLE-HS-L and TLE-HS-R, separately) from HC. While the performance of the latter model was higher than that of a pipeline trained on randomized data, the sensitivity and specificity, especially of DL models, were suboptimal. When classifying TLE patients based on their side of pathology (i.e., TLE-HS-L vs. TLE-HS-R), DL underperformed relative to SVM (77% ± 6.1% vs. 83% ± 3.6%, *p* = 0.97) likely driven by DL’s poorer sensitivity/specificity. As expected, ipsilateral hippocampal volumes had the highest influence on the prediction models in all cases. Other ROIs that showed consistently high importance were the contralateral amygdala, ipsilateral thalamus, and different portions of the bilateral lateral temporal regions and frontal regions.

### 3.2 Diffusion data

#### 3.2.1 Fractional anisotropy

**Fig. 3:**
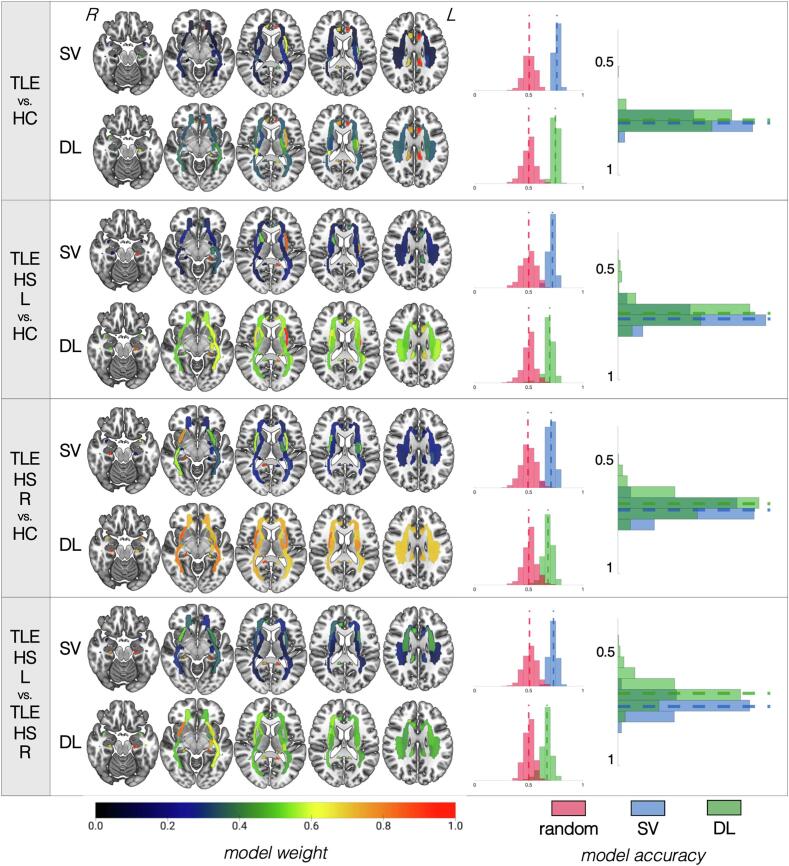
**Summary of results for models based on fractional anisotropy data for patients with hippocampal sclerosis.** For each of the four classifications (identified in the leftmost column), the top row shows the results for the support vector (SV) approach and the bottom row shows the results for the deep learning (DL) approach. White matter tracts are shown on axial slices from ventral to dorsal. The orientation corresponds to radiological reference (i.e., the left side of the axial slice corresponds to the right side of the brain, and vice versa). Each bundle corresponds to a region of interest (ROI) based on the Johns Hopkins University atlas colored based on the colormap at the bottom of the figure and normalized from 0 to 1, where higher values (more red regions) correspond to the most influential ROIs for that particular model. For each classification approach, a distribution of accuracies is shown to the right for permutated labels (red) or real labels (blue for SV, green for DL). The rightmost graph compares the accuracy of SV vs. DL against each other. Notice that models for which ROI weights have less variable distribution (i.e., all ROIs are relatively of equal importance for classification, as shown by a similar color range across all regions) tend to have worse performance accuracies. (For interpretation of the references to color in this figure legend, the reader is referred to the web version of this article.)

**Fig. 4:**
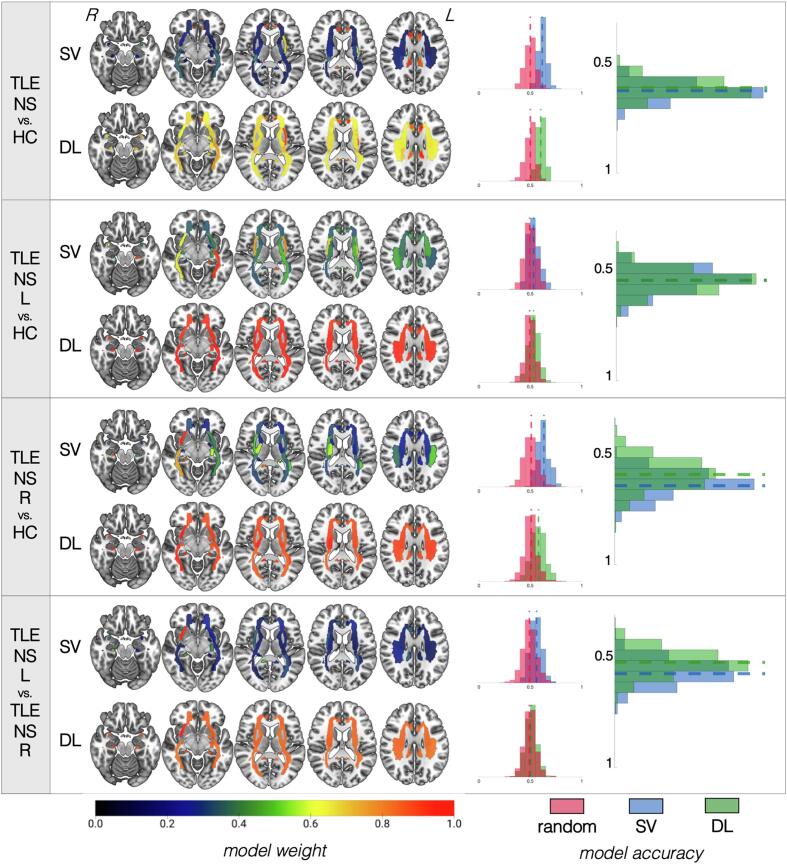
**Summary of results for models based on fractional anisotropy data for patients without hippocampal sclerosis.** For each of the four classifications (identified in the leftmost column), the top row shows the results for the support vector (SV) approach and the bottom row shows the results for the deep learning (DL) approach. White matter tracts are shown on axial slices from ventral to dorsal. The orientation corresponds to radiological reference (i.e., the left side of the axial slice corresponds to the right side of the brain, and vice versa). Each bundle corresponds to a region of interest (ROI) based on the Johns Hopkins University atlas colored based on the colormap at the bottom of the figure and normalized from 0 to 1, where higher values (more red regions) correspond to the most influential ROIs for that particular model. For each classification approach, a distribution of accuracies is shown to the right for permutated labels (red) or real labels (blue for SV, green for DL). The rightmost graph compares the accuracy of SV vs. DL against each other. Notice that models for which ROI weights have less variable distribution (i.e., all ROIs are relatively of equal importance for classification, as shown by a similar color range across all regions) tend to have worse performance accuracies. (For interpretation of the references to color in this figure legend, the reader is referred to the web version of this article.)

The performance of our classification pipelines based on FA data are summarized in Fig. 3 for patients with hippocampal sclerosis and in Fig. 4 for patients without hippocampal sclerosis, as well as **Supplementary Table 4** (for optimal pipeline parameters, please see **Supplementary Note 3).**

**Patients with hippocampal sclerosis**: In all comparisons of TLE groups vs. HC, the SVM and DL pipelines demonstrated no detectable differences in classification accuracy. There was a 74 ± 2.7% (DL) to 76 ± 5.4% (SVM) accuracy to correctly distinguish all patients with TLE-HS from HC based on FA values. Similar accuracies (71–72%) were found when the models attempted to classify TLE patients based on the specific side of presumed seizure onset (i.e., TLE-HS-L and TLE-HS-R, separately) from HC. This model showed significantly higher sensitivity/specificity than a classifier trained on randomized data for SVM but not for DL. Lateralization of TLE-HS patients demonstrated 73% ± 3.4% accuracy with SVM and 66% ± 5.2% with DL, demonstrating the relative suboptimal performance of the latter (p = 0.97), again driven by the lower sensitivity/specificity. In all cases, the bilateral (ipsilateral > contralateral) parahippocampal cingulate bundles had the most weight. The external capsule appeared to have more prominent weight for classification of TLE groups vs. HC than for lateralization.

**Patients without hippocampal sclerosis:** In all cases, SVM and DL pipelines demonstrated no significant differences in classification accuracy (*p* = 0.44 to 0.83). There was a 60 ± 4% (DL) to 62% ± 4% (SVM) accuracy to correctly distinguish all patients with TLE-NS from HC based on FA values. The performance was lower (53–63%) when the models attempted to classify TLE patients based on the specific side of presumed seizure onset (i.e., TLE-NS-L and TLE-NS-R, separately) from HC, and these models were not significantly more accurate than models trained on randomized data. Similarly, attempts to predict lateralization of TLE-NS patients demonstrated low accuracy (51% DL, 56% SVM) which did not outperform models trained on randomized data. The bilateral cingulate bundles showed the most prominence for SVM and DL models classifying all TLE-NS independently of side vs. HC, but these tracts were not as important for other models. The ipsilateral sagittal stratum was important in classifying TLE-NS patients from each side against controls. For TLE-NS-R vs. HC in particular, the ipsilateral cingulate, contralateral fornix, and ipsilateral inferior fronto-occipital fasciculus, as well as the splenium of the corpus callosum, all appeared to influence the SVM model more heavily than for other comparisons.

#### 3.2.2 Radial diffusivity

**Fig. 5:**
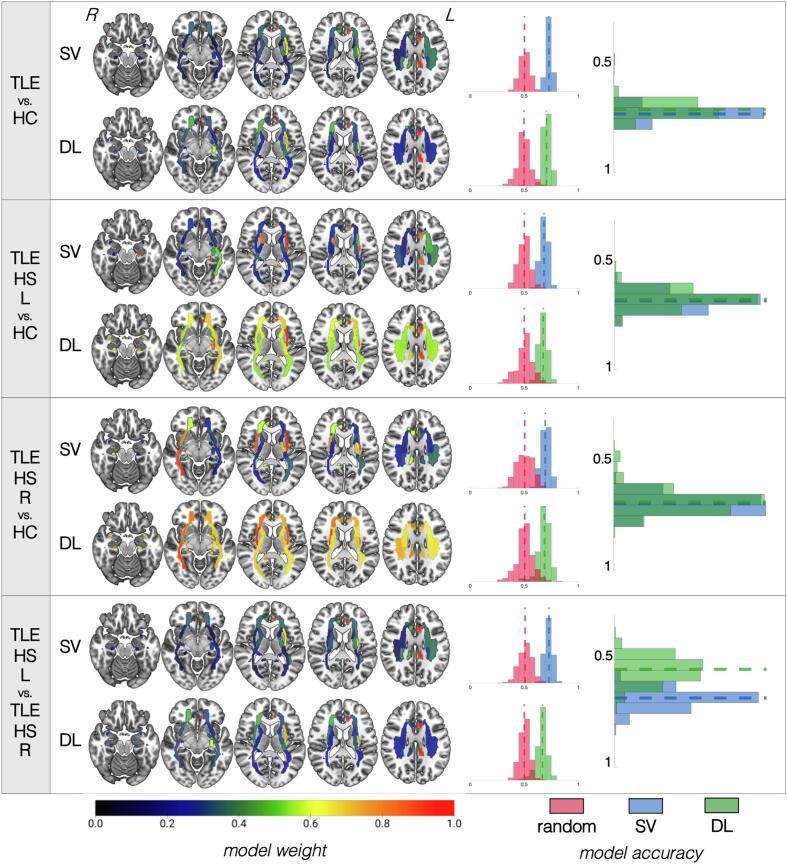
**Summary of results for models based on radial diffusivity data for patients with hippocampal sclerosis.** For each of the four classifications (identified in the leftmost column), the top row shows the results for the support vector (SV) approach and the bottom row shows the results for the deep learning (DL) approach. White matter tracts are shown on axial slices from ventral to dorsal. The orientation corresponds to radiological reference (i.e., the left side of the axial slice corresponds to the right side of the brain, and vice versa). Each bundle corresponds to a region of interest (ROI) based on the Johns Hopkins University atlas colored based on the colormap at the bottom of the figure and normalized from 0 to 1, where higher values (more red regions) correspond to the most influential ROIs for that particular model. For each classification approach, a distribution of accuracies is shown to the right for permutated labels (red) or real labels (blue for SV, green for DL). The rightmost graph compares the accuracy of SV vs. DL against each other. Notice that models for which ROI weights have less variable distribution (i.e., all ROIs are relatively of equal importance for classification, as shown by a similar color range across all regions) tend to have worse performance accuracies. (For interpretation of the references to color in this figure legend, the reader is referred to the web version of this article.)

**Fig. 6:**
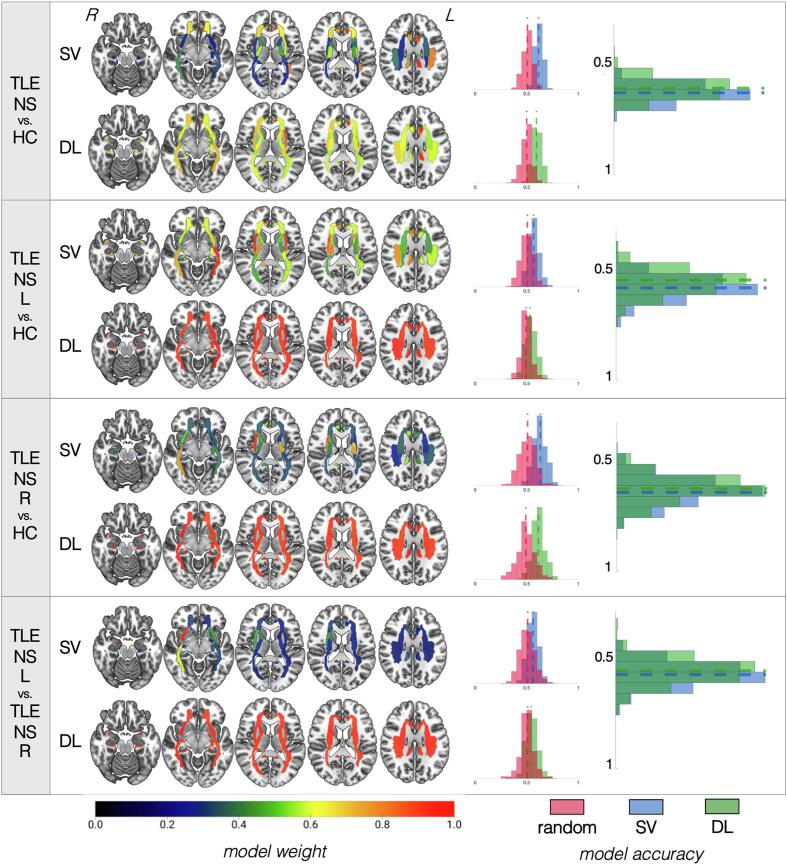
**Summary of results for models based on radial diffusivity data for patients without hippocampal sclerosis.** For each of the four classifications (identified in the leftmost column), the top row shows the results for the support vector (SV) approach and the bottom row shows the results for the deep learning (DL) approach. White matter tracts are shown on axial slices from ventral to dorsal. The orientation corresponds to radiological reference (i.e., the left side of the axial slice corresponds to the right side of the brain, and vice versa). Each bundle corresponds to a region of interest (ROI) based on the Johns Hopkins University atlas colored based on the colormap at the bottom of the figure and normalized from 0 to 1, where higher values (more red regions) correspond to the most influential ROIs for that particular model. For each classification approach, a distribution of accuracies is shown to the right for permutated labels (red) or real labels (blue for SV, green for DL). The rightmost graph compares the accuracy of SV vs. DL against each other. Notice that models for which ROI weights have less variable distribution (i.e., all ROIs are relatively of equal importance for classification, as shown by a similar color range across all regions) tend to have worse performance accuracies. (For interpretation of the references to color in this figure legend, the reader is referred to the web version of this article.)

The performance of our classification pipelines based on RD are summarized in Fig. 5 for patients with hippocampal sclerosis and Fig. 6 for patients without hippocampal sclerosis, as well as **Supplementary Table 5** (for optimal pipeline parameters, please see **Supplementary Note 4).**

**Patients with hippocampal sclerosis**: In all TLE vs. HC comparisons, SVM and DL pipelines demonstrated no significant differences in classification accuracy. There was a 71 ± 5.9% (DL) to 73 ± 2.5% (SVM) accuracy for identifying all patients with TLE-HS versus HC based on RD values. Slightly lower accuracies (67–69%) were found when the models attempted to classify TLE patients based on the specific side of presumed seizure onset (i.e., TLE-HS-L and TLE-HS-R, separately) from HC. These models showed significantly higher sensitivity/specificity than a classifier trained on randomized data for SVM but not for DL. Lateralization of TLE-HS patients demonstrated 68% ± 4.1% accuracy with SVM but only 55% ± 5.7% with DL, which had relatively suboptimal performance (p = 1.0), again driven by the lower sensitivity/specificity of the latter. In all cases, the bilateral (ipsilateral > contralateral) cingulate bundles had the most weight. The external capsule appeared to have a more prominent weight for classification of TLE groups vs. HC than for lateralization. For lateralization in particular, the superior corona radiata bilaterally demonstrated higher weights than for TLE vs. HC comparisons. Bilateral inferior fronto-occipital fasciculi were particularly influential for the SVM model of TLE-HS-R vs. HC classification.

**Patients without hippocampal sclerosis:** In all cases, SVM and DL pipelines demonstrated no significant differences in classification accuracy. There was a 59 ± 4.5% (DL) to 62% ± 4% (SVM) accuracy to correctly identify all patients with TLE-NS from HC based on RD values. The performance was lower (54–63%) when the models attempted to classify TLE patients based on the specific side of presumed seizure onset (i.e., TLE-HS-L and TLE-HS-R, separately) from HC, which were not significantly more accurate than models trained on randomized data. Similarly, attempts to classify the lateralization of TLE-NS patients demonstrated low accuracy (55% DL, 56% SV) which was not better than chance. Bilateral cingulate bundles showed the most prominence for SVM and DL models classifying all TLE-NS independently of side vs. HC, but these tracts were not as important for other models. The ipsilateral sagittal stratum was important in classifying TLE-NS patients from each side against controls. Bilateral (ipsilateral > contralateral) cingulum and external capsule bundles contributed heavily to all classification models. The inferior fronto-occipital fasciculus was more important particularly for TLE-NS-R both vs. TLE-NS-L and also vs. HC. The sagittal stratum was influential for comparisons against HC ipsilaterally, and bilaterally for lateralization.

## 4 Discussion

This study leveraged the large dataset from the ENIGMA-Epilepsy consortium to evaluate the performance of conventional machine learning (SVM) and DL in distinguishing patients with TLE versus healthy controls, as well as lateralizing TLE using individual structural and diffusion data at an ROI level. The overarching goal of the study was to demonstrate the feasibility of applying machine learning and artificial intelligence frameworks to large cohorts of clinically-obtained imaging data. We observed that 1) SVM was more consistently accurate than DL in outperforming random models, 2) models to discriminate TLE patients from healthy controls performed better than models to lateralize the seizure focus in TLE patients, 3) structural and diffusion-based models showed overall similar classification accuracies, 4) classification models of patients with HS were more accurate than models aimed at stratifying non-lesional patients, and 5) many ipsilateral and contralateral white matter tracts contributed to model classifications.

### 4.1 ROI level data

While this is the largest artificial intelligence study of quantitative imaging in epilepsy, our study was based on ROI-level data. Specifically, for each subject, there were 152 data points for structural imaging (derived from 84 ROIs) and 41 data points for diffusion imaging, all representing the average values within each of the ROIs. This is a much lower resolution compared to voxel-wise data, in which each subject would have millions of data points per imaging modality. The motivations for conducting an ROI-level artificial intelligence study are: 1) the available ROI-level ENIGMA dataset yields a substantially larger sample size than single or even multi-site cohorts; 2) the identification of promising (statistically significant, i.e., better than chance) results with ROI-level data motivates future studies with higher-resolution data; and 3) we can find out if ROI-level analysis of large sample sizes is sufficient for near-perfect classification; in other words, it can help evaluate if the determinant for classification accuracy in machine learning models is merely sample size.

We observed promising classification accuracies for many imaging modalities and metrics (detailed below). They were, however, not perfect. As such, it is possible to derive two initial conclusions: 1) artificial intelligence has a growing potential in the field of epilepsy, and 2) further refinements will likely not depend on larger sample sizes of ROI-level data, but on higher resolution imaging, including voxel- and vertex-level data.

### 4.2 SVM vs. DL model performance

SVM and DL showed similar accuracy for most models stratifying patients from healthy controls. However, several SVM-based models performed better in terms of sensitivity and specificity relative to models trained on randomized data, whereas DL-based models for the same imaging modalities showed non-superior performance. In addition, there was overall relative suboptimal DL performance relative to SVM particularly for lateralization of TLE groups across imaging modalities. SVM models may be moderately reliable for classification and lateralization of TLE based on structural and diffusion sequences, as previously shown by other smaller-scale studies using modalities such as resting state functional MRI (Zhang et al., 2012).

One reason for DL’s lower performance might be related to the type of features used in our analyses. The computation of whole-ROI averages from multiple voxels can be regarded as a coarse approach with information inherently being lost. This approach may be suboptimal for DL techniques as these can make use of fine-grained features such as edges or clusters within a complete dataset that has not been aggregated into a fixed set of ROIs. In such cases, the performance of a simple linear decision boundary found by a support vector classification technique is likely to be just as accurate, or possibly more accurate, than a complex decision network found by a deep learning approach. These findings highlight the need to enter different types of features with varying degrees of resolution (i.e. fine-grained for DL and course-grained for SV).

### 4.3 TLE diagnosis vs. Lateralization

Our machine learning models were similar in distinguishing TLE patients from healthy controls and were less reliable in lateralizing patients based on their side of TLE, particularly for SVM. Consistently across SVM and DL, however, when classifying non-lesional patients, the models performed slightly worse for lateralization than diagnostic stratification. Structural-based models had a superior accuracy for detection over lateralization of TLE, particularly for SVM (83% detection vs. 67% lateralization, on average). The issue of epilepsy lateralization was one of the earliest to be addressed by machine learning approaches with modalities including positron emission tomography (PET) (Lee et al., 2000), photon emission computed tomography (SPECT) (Lopes et al., 2010), and diffusion magnetic resonance techniques (Davoodi-Bojd et al., 2016) including diffusion tensor (Ahmadi et al., 2009, Concha et al., 2012, Kamiya et al., 2016) and kurtosis (Del Gaizo et al., 2017) imaging, with mean accuracies overall similar to those found in this study. Future studies might enhance lateralization accuracy by building classifiers based on multi-modal features, particularly for non-lesional patients, whose lateralization was suboptimal and often not better than that of a random model.

### 4.4 Structural vs. diffusion models

Models trained with features derived from structural and diffusion imaging performed relatively similarly. One exception was, as stated above, noticeably higher accuracy of structural than diffusion models for lateralization of lesional patients. Both types of modalities provide valuable information for classification, but multimodal approaches may enhance model performance. A combination of data types could be explored both “within modality” (for example, combining FA, RD, MD, and other diffusion scalar metrics) but also “across modalities” (for example, combining regional volumetrics with FA, etc). In this study, diffusion data obtained at the ROI-level condensed large amounts of white matter bundle information into regional data points. Utilizing tractography metrics that do not conform to predefined white matter bundles (i.e., ROI-to-ROI connectivity) might also yield higher accuracies by providing higher resolution at the whole-brain level, unmasking patterns of abnormal network organization undetected by nodal measures. Similarly, future studies could combine features derived from graph theory metrics or other connectome-based approaches, which could serve as topological biomarkers for the detection and lateralization of TLE.

### 4.5 Lesional vs. non-lesional patients

As expected, models performed better in lesional than non-lesional patients. The expectation of machine learning models with so-called ‘MRI-negative’ patients is that these computational techniques will be able to detect features otherwise visually ignored or undetectable by human experts to enhance classification accuracy. We did not have access to non-lesional patients for structural data, so our interpretation must be taken with caution. However, based on the diffusion models, it is evident that regional information may be suboptimal to detect and lateralize non-lesional patients, with accuracies mostly in the 53–63% range. This is in line with previous studies in focal cortical dysplasia showing similar accuracy (58%) in detecting focal cortical dysplasia lesions in MRI studies deemed to be ‘negative’ by human experts (Ahmed et al., 2015, Hong et al., 2014). Importantly, we must acknowledge that there is a bias inherent to the proportion of lesional vs. non-lesional patients in adults with TLE, which invariably features a larger sample of the former group. A larger number of patients has the potential to allow for more fine-grained modeling based on more samples for training. Future studies could address this issue by testing the accuracies of samples of similar size in each group.

### 4.6 Model features

Across different models, the features with maximal importance for classification accuracy included structures in the limbic region (e.g., cingulate bundles and sagittal stratum) as well as extra-limbic portions. In structural models, ipsilateral hippocampal volumes had the highest weight with variable influence of other medial temporal structures, such as the amygdala, as well as lateral temporal regions and extra temporal areas (including the thalamus), all of which have been shown to have volume abnormalities using *meta*-analytical group comparisons (Whelan et al., 2018). Similarly, diffusion models demonstrated that the most influential white matter ROIs were along limbic (e.g., cingulate bundles, sagittal stratum) and extra-limbic (e.g., external capsule) sub-networks, aligned with recent mega-analysis across epilepsy syndromes by the ENIGMA-Epilepsy consortium (Hatton et al., 2020). These findings highlight the now well-established observation that plastic changes in focal epilepsy occur beyond the presumed area of seizure focus, stressing the nature of epilepsy as a disorder characterized by aberrant brain network reorganization (Engel et al., 2013, Gleichgerrcht and Bonilha, 2017, Richardson, 2012, Tavakol et al., 2019).

**Limitations**

The initial cohort of ENIGMA-Epilepsy patients included ROI-based data only. Because post-processing was performed at each site following structured guidelines and quality checks from the consortium protocols, the data collected were exclusively at the ROI level. We believe that the suboptimal performance of some of the classification models reported above is being driven by the input data existing at the ROI level, which summarizes potentially heterogeneous information into a single value. The trade-off to this limitation, however, was the large volume of patients with TLE from epilepsy centers across the world. One alternative approach in the absence of voxel-level data would be to implement finer resolution atlases that yield smaller ROIs, hence counting on a more rich dataset per patient where each individual value reflects a more constrained anatomical region. As such, this large multi-site, multi-nationality representation of TLE provides the most comprehensive representation of the disease to date. Nonetheless, such a large database cohort carries the potential to be affected by confounders intrinsic to the retrospective nature of our design, including variations in type of scanner, variability in local quality assurance protocols, acquisition protocols, and regional clinical diagnostic pipelines.

We addressed imaging heterogeneity by applying a well-established data harmonization technique validated in diffusion tensor imaging data (Fortin et al., 2017) and applied it to multi-site cohorts of epilepsy patients (Hatton et al., 2020). By decreasing imaging data heterogeneity, we believe that the results from this study may be more generalizable. However, there are a few limitations to this approach. First, because ComBat is applied to the entire data set, and then split into a train, validation, and test dataset, a bias is likely introduced into the classification pipeline. More specifically, because the harmonization parameters learned by ComBat included the test data, the test data is not completely unseen which may slightly enhance classification performance. Second, ROI data from the same site may appear in both the training and test data set, so a site bias may be introduced into the classification. However, since ComBat was applied to the entire dataset prior to pipeline construction, multi-site differences were minimized (as best as possible) and classification performance enhancements due to site bias were likely mitigated. More specifically, relatively lower classification accuracy (i.e. 50 to 70%) could be an indicator of site bias removal.

In addition, it is important to consider other non-imaging related sources that may further limit model accuracy. For instance, the overall underperformance of classification models for lateralization of TLE may be negatively influenced by how left vs. right disease is determined (i.e., based on EEG and/or imaging findings). However, future studies could use a different approach by comparing accuracies exclusively among those who achieved seizure freedom (i.e., Engel class I or ILAE 1 outcome), a proxy for “gold standard” confirmation of laterality (for further discussion, see Liu et al., 2016).

The limitations of this study also highlight the importance of multimodal approaches. Because data collection for ENIGMA-Epilepsy occurred in different waves for structural and diffusion images, we were not able to cross-reference anonymized data across cohorts in order to test bimodal inputs to train the classifier. Efforts are currently ongoing for each consortium to identify the overlap between structural and diffusion cohorts to enable multimodal testing. In addition, ENIGMA-Epilepsy also includes patients with generalized genetic epilepsy and extratemporal focal epilepsy. Similar studies with these populations may highlight classifier accuracy drivers common for all epilepsy syndromes and also distinct patterns that inform on syndrome-specific characteristics.

## 5 Conclusions

Artificial intelligence might allow for the classification and lateralizing of TLE using ROI-level data, with moderate accuracy. Cortical thickness and surface area were equivalent in accuracy compared with fractional anisotropy and radial diffusivity, and classification was better among patients with hippocampal atrophy. DL was not superior to SVM at ROI-level data. Extra-hippocampal features were important in all classification models, suggesting that machine learning can use information beyond regions that are visually identified in human qualitative analyses.

These results suggest that machine learning approaches can aid in the classification of TLE (which could potentially aid in radiological diagnosis). This process may be improved by employing higher resolution imaging data (i.e., pre-processed images with no smoothing or manipulations) and multimodal approaches. This study will be a driver for ENIGMA-Epilepsy and other large data consortia to centralize and harmonize raw data for machine learning analyses.

## 6 Funding and acknowledgements

B.C.M. is supported by NINDS R21 NS107739-01A1. M.K.M.A. is supported by FAPESP 15/17066-0. A.B. is supported by CIHR MOP-57840. N.Be. is supported by CIHR MOP-123520; CIHR MOP-130516. B.Ber. acknowledges research support from NSERC (Discovery-1304413), CIHR (FDN-154298), Azrieli Center for Autism Research of the Montreal Neurological Institute (ACAR), SickKids Foundation (NI17-039), and salary support from FRQS (Chercheur Boursier Junior 1). L.C. is supported by Mexican Council of Science and Technology (CONACYT 181508, 232676, 251216, and 280283); UNAM-DGAPA (IB201712). O.D. is supported by Finding A Cure for Epilepsy and Seizures (FACES). J.S.D. is supported by NIHR.

N.K.F. is supported by DFG FO750/5-1. R.Kä. is supported by Saastamoinen Foundation.

S.S.K. is supported by Medical Research Council (MR/S00355X/1 and MR/K023152/1) and Epilepsy Research UK (1085). P.K. is supported by S10OD023696; R01EB015611.

S.La. is funded by CIHR. P.M. was supported by the PATE program (F1315030) of the University of Tübingen. S.M. is supported by Italian Ministry of Health funding grant NET-2013-02355313. T.J. is supported by NHMRC Program Grant. M.R. is supported by Medical Research Council programme grant (MR/K013998/1); Medical Research Council Centre for Neurodevelopmental Disorders (MR/N026063/1); NIHR Biomedical Research Centre at South London and Maudsely NHS Foundation Trust. R.R.-C. is supported by the Fonds de recherche du Québec – Santé (FRQS-291486). D.J.S. is supported by SA Medical Research Council. Work developed within the framework of the DINOGMI Department of Excellence of MIUR 2018–2022 (legge 232 del 2016). R.H.T. is supported by Epilepsy Research UK. C.L.Y. is supported by FAPESP - BRAINN (2013/07599-3); CNPQ (403726/2016-6). J.S.Z. is supported by National Nature Science Foundation of China (No. 61772440). C.R.M. is supported by NIH R01 NS065838; R21 NS107739. L.B. is supported by R01NS110347​ (NIH/NINDS). A.A. holds an MRC eMedLab Medical Bioinformatics Career Development Fellowship; this work was partly supported by the Medical Research Council [grant number MR/L016311/ 1]. R.W. received support from the Swiss League Against Epilepsy. Core funding for ENIGMA was provided by the NIH Big Data to Knowledge (BD2K) program under consortium grant U54 EB020403. The Bern research centre was funded by Swiss National Science Foundation (grant 180365). This work was partly undertaken at UCLH/UCL, which received a proportion of funding from the Department of Health’s NIHR Biomedical Research Centres funding scheme. The work was also supported by the Epilepsy Society, UK. We are grateful to the Wolfson Trust and the Epilepsy Society for supporting the Epilepsy Society MRI scanner. The UNICAMP research centre was funded by FAPESP (São Paulo Research Foundation); Contract grant number: 2013/07559-3. Lastly, we are grateful for software development and high-performance computing work performed by UNC Chapel Hill graduate research assistant Kyuyeon Kim.

## Credit Author Statement

Data curation: N.Ba., A.B., N.Be., L.B., E.G., F.C., C.De., O.D., M.D., N.K.F., A.G., R.G., R.Kä., S.S.K., P.K., A.L., S.L., C.R.M., S.M., T.J., H.R.P., M.P.R., T.R., S.M.S., H.S.-Z., P.S., B.W., R.W., Y.Y., J.Z.

Formal analysis: E.G., L.B., S.N.H., P.K., C.R.M., B.C.M.

Methodology: E.G., B.C.M., L.B., S.A., B.Ben., M.E.C., M.D., S.N.H., S.S.K., R.Ko., B.A.K.K., M.L., P.M., C.R.M., H.R.P., J.C.P., R.R.-C., B.S., P.N.T., A.E.V., L.V., C.L.Y.

Writing – original draft: L.B., E.G., C.R.M., B.C.M.

Writing – reviewing & editing: S.A., A.A., B.Ben., A.B., N.Be., B.Ber., L.B., M.E.C., F.C., L.C., M.D., J.S.D., N.K.F., M.G., A.G., E.G., B.G., S.S.K., P.K., B.A.K.K., S.L., S.La., M.L., E.L., M.M., C.R.M., B.C.M., T.J., H.R.P., M.P.R., S.M.S., D.J.S., P.S., S.I.T., P.M.T., L.V., F.v.P., S.B.V.

Project administration: C.R.M., S.M.S.

## Declaration of Competing Interest

The authors declare the following financial interests/personal relationships which may be considered as potential competing interests: B.Ben. is the cofounder of AIRAmed GmbH, a company that offers brain segmentation. N.K.F. holds Honoraria from Bial, Eisai, Philips/EGI, UCB. D.J.S. has received research grants or consultance honoraria from Johnson & Johnson, Lundbeck, Servier, and Takeda. P.S. received speaker fees from and is on advisory boards for Biomarin, Zogenyx, GW Pharmaceuticals; research funding by ENECTA BV, GW Pharmaceuticals, Kolfarma srl., Eisai. P.M.T. received a research grant from Biogen, Inc., and was a paid consultant for Kairos Venture Capital, Inc., USA, for projects unrelated to this work. M.G. received fees and travel support from Bial Pharmaceutical and Netlé Health Science outside the submitted work.

## Footnotes

1 http://enigma.ini.usc.edu/ongoing/dti-working-group/

2 https://www.nitrc.org/projects/enigma_dti/
