# Supplementary material for: Artificial intelligence for classification of temporal lobe epilepsy with ROI-level MRI data: A worldwide ENIGMA-Epilepsy study
Source: Neuroimage Clin. 2021 Jul 24;31:102765. doi: 10.1016/j.nicl.2021.102765 (PMC8346685; doi:10.1016/j.nicl.2021.102765)
Supplement: Supplementary data 3 [file mmc3.docx]

**Supplemental Information**

**Supplementary Notes**

**Supplementary Note 1. Deep learning classifier (DLC) design**

The DLC model is represented by a dense neural network design (**Supplementary Figure 2**). To compute the edge weight and bias values at each layer that resulted in the highest classification accuracy the DLC model was optimized using the Adadelta adaptive learning-rate method [S1], model checkpointing [S2] based on validation data set classification accuracy (every 10 epochs), and a back-propagation technique that used a stochastic gradient decent algorithm [S3] and the categorical cross-entropy loss function [S4]. In general, the network architecture included one visible (input) layer that defined N=152 nodes when structural ROI data was used (**Manuscript Section 2.2**: Structural imaging dataset) and N=41 nodes when diffusion ROI data was used (**Manuscript Section 2.3**: Diffusion data set), two dense hidden layers (L1 and L2), two hidden activation layers (RELU1 and RELU2), one hidden $l_{2}$ regularization layer, one hidden dropout layer (DO1), and one output layer. The two hidden activation layers used the rectified linear unit (ReLU) functions, and the output layer has one node and implements the softmax function. To mitigate overfitting the dropout [S5] and $l_{2}$regularization layers were included in the design.

An exhaustive grid search procedure (**Supplemental:** Section V) found the: optimal number of nodes in hidden layer one (L1) and hidden layer two (L2); the optimal hidden layer one $l_{2}$regularization penalty; and the optimal percent of nodes to randomly remove in the hidden dropout layer. One additional supervised training learning layer was added to train the DLC model that defined one node which represented the classification label (e.g. HC or TLE). Once the supervised training step completed, the supervised training layer was removed, and the one node in the output layer was used for classification. In our training approach, the DLC model was ran for 500 epochs and the DLC model checkpoint that yielded the highest validation classification accuracy was determined to be the highest performing model that was applied to the test data set. The software used to develop, train, and test the dense neural network is written in Python, and used the publicly available Theano (http://deeplearning.net/software/theano/) and Keras (https://keras.io/) Python deep learning libraries that wrap the C++ NVIDIA CUDA deep neural network libraries (https://developer.nvidia.com/cudnn). All the reported results were executed on a Tesla V100-SXM2 GPUs with 5120 NVIDIA CUDA cores and 16 GB HBM2 memory (<https://its.unc.edu/research-computing/techdocs/getting-started-on-longleaf/#System%20Information>)

**Supplementary Note 2. Structural ROI grid search results**

HC vs. TLE-HS: The optimal DLC model parameters found by the grid search were hidden layer one nodes=10, hidden layer two nodes units=5, hidden layer one $l_{2}$ regularization=0.2, and hidden layer one drop-out rate=0.3. The optimal SVC regularization penalty found by the grid search was C=0.1.

HC vs. TLE-HS-L: The optimal DLC model parameters found by the grid search were hidden layer one nodes=10, hidden layer two nodes units=5, hidden layer one $l_{2}$ regularization=0.2, and hidden layer drop-out rate=0.3. The optimal SVC regularization penalty found by the grid search was C=0.1.

HC vs. TLE-HS-R: The optimal DLC model parameters found by the grid search were hidden layer one nodes=10, hidden layer two nodes units=7, hidden layer one $l_{2}$ regularization=0.1, and hidden layer drop-out rate=0.3. The optimal SVC regularization penalty found by the grid search was C=0.1.

TLE-HS-L vs. TLE-HS-R: The optimal DLC model parameters found by the grid search were hidden layer one nodes=10, hidden layer two nodes units=3, hidden layer one $l_{2}$ regularization=0.1, and hidden layer drop-out rate=0.2. The optimal SVC regularization penalty found by the grid search was C=0.1.

**Supplementary Note 3. Fractional anisotropy (FA) diffusion ROI grid search results**

HC vs. TLE-HS: The optimal DLC model parameters found by the grid search were hidden layer one nodes=20, hidden layer two nodes units=5, hidden layer one $l_{2}$ regularization=0.2, and hidden layer drop-out rate=0.2. The optimal SVC regularization penalty found by the grid search was C=0.25.

HC vs. TLE-HS-L: The optimal DLC model parameters found by the grid search were hidden layer one nodes=20, hidden layer two nodes units=3, hidden layer one $l_{2}$ regularization=0.2, and hidden layer drop-out rate=0.2. The optimal SVC regularization penalty found by the grid search was C=0.1.

HC vs. TLE-HS-R: The optimal DLC model parameters found by the grid search were hidden layer one nodes=10, hidden layer two nodes units=3, hidden layer one $l_{2}$ regularization=0.1, and hidden layer drop-out rate=0.3. The optimal SVC regularization penalty found by the grid search was C=0.1.

TLE-HS-L vs. TLE-HS-R: The optimal DLC model parameters found by the grid search were hidden layer one nodes=10, hidden layer two nodes units=5, hidden layer one $l_{2}$ regularization=0.1, and hidden layer drop-out rate=0.2. The optimal SVC regularization penalty found by the grid search was C=1.1.

HC vs. TLE-NS: The optimal DLC model parameters found by the grid search were hidden layer one nodes=20, hidden layer two nodes units=7, hidden layer one $l_{2}$ regularization=0.2, and hidden layer drop-out rate=0.2. The optimal SVC regularization penalty found by the grid search was C=0.1.

HC vs. TLE-NS-L: The optimal DLC model parameters found by the grid search were hidden layer one nodes=10, hidden layer two nodes units=3, hidden layer one $l_{2}$ regularization=0.2, and hidden layer drop-out rate=0.2. The optimal SVC regularization penalty found by the grid search was C=0.1.

HC vs. TLE-NS-R: The optimal DLC model parameters found by the grid search were hidden layer one nodes=20, hidden layer two nodes units=7, hidden layer one $l_{2}$ regularization=0.1, and hidden layer drop-out rate=0.3. The optimal SVC regularization penalty found by the grid search was C=0.1.

TLE-HS-L vs. TLE-NS-R: The optimal DLC model parameters found by the grid search were hidden layer one nodes=20, hidden layer two nodes units=5, hidden layer one $l_{2}$regularization=0.1, and hidden layer drop-out rate=0.2. The optimal SVC regularization penalty found by the grid search was C=0.1.

**Supplementary Note 4. Radial diffusivity (RD) diffusion ROI grid search results**

HC vs. TLE-HS: The optimal DLC model parameters found by the grid search were hidden layer one nodes=10, hidden layer two nodes units=5, hidden layer one $l_{2}$ regularization=0.2, and hidden layer drop-out rate=0.2. The optimal SVC regularization penalty found by the grid search was C=1.5.

HC vs. TLE-HS-L: The optimal DLC model parameters found by the grid search were hidden layer one nodes=20, hidden layer two nodes units=5, hidden layer one $l_{2}$ regularization=0.1, and hidden layer drop-out rate=0.3. The optimal SVC regularization penalty found by the grid search was C=0.5.

HC vs. TLE-HS-R: The optimal DLC model parameters found by the grid search were hidden layer one nodes=10, hidden layer two nodes units=3, hidden layer one $l_{2}$ regularization=0.1, and hidden layer drop-out rate=0.2. The optimal SVC regularization penalty found by the grid search was C=0.1.

TLE-HS-L vs. TLE-HS-R: The optimal DLC model parameters found by the grid search were hidden layer one nodes=10, hidden layer two nodes units=5, hidden layer one $l_{2}$ regularization=0.1, and hidden layer drop-out rate=0.3. The optimal SVC regularization penalty found by the grid search was C=1.5.

HC vs. TLE-NS: The optimal DLC model parameters found by the grid search were hidden layer one nodes=10, hidden layer two nodes units=7, hidden layer one $l_{2}$ regularization=0.1, and hidden layer drop-out rate=0.2. The optimal SVC regularization penalty found by the grid search was C=0.7.

HC vs. TLE-NS-L: The optimal DLC model parameters found by the grid search were hidden layer one nodes=20, hidden layer two nodes units=3, hidden layer one $l_{2}$ regularization=0.1, and hidden layer drop-out rate=0.2. The optimal SVC regularization penalty found by the grid search was C=0.1.

HC vs. TLE-NS-R: The optimal DLC model parameters found by the grid search were hidden layer one nodes=20, hidden layer two nodes units=7, hidden layer one $l_{2}$ regularization=0.1, and hidden layer drop-out rate=0.2. The optimal SVC regularization penalty found by the grid search was C=2.0.

TLE-HS-L vs. TLE-NS-R: The optimal DLC model parameters found by the grid search were hidden layer one nodes=20, hidden layer two nodes units=3, hidden layer one $l_{2}$ regularization=0.2, and hidden layer drop-out rate=0.2. The optimal SVC regularization penalty found by the grid search was C=0.1.

**Supplementary Figure Captions**

***Supplementary Figure 1.*** Configurable pipeline approach. (**A**) Basic description and operation of the proposed software pipeline. (**B**) Any model in the pipeline can be replaced by a different model with little effort, making our approach configurable (e.g. replace SV classification model with DL one). Of note, harmonization and imbalance correction was not included in our pipeline as these were accomplished prior to pipeline construction.

***Supplementary Figure 2.*** Dense neural network architecture that defines one visible layer (input layer with N nodes), two hidden dense layers (L1 and L2), one hidden $l_{2}$ regularization layer, one hidden dropout layer (DO1), two hidden activation layers (RELU1 and RELU2), and one output layer that includes one node.

**Supplemental References**

S1. Zeiler, Matthew D. Adadelta: an adaptive learning rate method., 2012, *arXiv preprint arXiv:1212.5701* (2012).

S2. Moshkov, N., Mathe, B., Kertesz-Farkas, A. *et al.* Test-time augmentation for deep learning-based cell segmentation on microscopy images. *Sci Rep* **10,**5068 (2020). https://doi.org/10.1038/s41598-020-61808-3

S3. SBottou, L., Stochastic gradient learning in neural networks. Proceedings of Neuro-Nımes, 1991. 91(8).

S4. Joe, H., Relative entropy measures of multivariate dependence. Journal of the American Statistical Association, 1989. 84(405): p. 157-164.

S5. Srivastava, N., et al., Dropout: A Simple Way to Prevent Neural Networks from Overfitting. Journal of Machine Learning Research, 2014. 15: p. 1929-1958.
