# Supplementary material for: Artificial intelligence for classification of temporal lobe epilepsy with ROI-level MRI data: A worldwide ENIGMA-Epilepsy study
Source: Neuroimage Clin. 2021 Jul 24;31:102765. doi: 10.1016/j.nicl.2021.102765 (PMC8346685; doi:10.1016/j.nicl.2021.102765)
Supplement: Supplementary data 4 [file mmc4.docx]

**Supplementary Table 1.** Structural imaging acquisition and processing details by research site.

| Site | Sequence | Field Strength | Acquisition Direction | # of Slices | Spacing between slices (mm) | Voxel Size  (mm^3^) | TI  (ms) | TE  (ms) | TR  (ms) | Flip Angle |
| --- | --- | --- | --- | --- | --- | --- | --- | --- | --- | --- |
| Bern | 3D T1 MPRAGE | 3 T Siemens Trio | Sagittal | 176 | 0 | 1 | 900 | 2.01 | 1500 | 9 |
| Bonn | 3D T1 MPRAGE | 3T Trio, Siemens | Coronal | 160 | 0 | 1 | 650 | 3.97 | 1300 | 10 |
| BRI | 3D T1 MPRAGE | 3T TrioTim Siemens | Sagittal | 192 | 0 | 0.9 | 900 | 2.6 | 1900 | 9 |
| Brussels | sense 3DT1TFE  s3DT1TFE | 1.5T Siemens  3.0T Siemens | Sagittal  Sagittal | 130  160 | 0  0 | 1.3*  1.3* | 880  1040 | 4.2  4.6 | 8.8  9.8 | 8  8 |
| CUBRIC | 3D T1 FSPGR | 3T GE HDx | Axial | 176 | 0 | 1 | 450 | 3 | 7.9 | 20 |
| EKUT_A | 3D T1 MPRAGE | 3T Siemens Skyra | Sagittal | 192 | 0 | 0.9 | 900 | 2.32 | 2300 | 8 |
| EKUT_B | 3D T1 MPRAGE | 3T Siemens TrioTim | Sagittal | 176-192 | 0 | 1 | 1100 | 3.03 | 2300 | 8 |
| EPICZ | 3D T1 FSPGR | 3 T Discovery MR750 GE | Sagittal | 184 | 0 | 1 | 650 | 3.7 | 9.2 | 12 |
| EPIGEN_3.0 | 3D T1 TFES | 3 T Philips Achieva | Sagittal | 160 | 0 | 1 | - | 3.9 | 8.5 | 8 |
| EPIGEN_1.5 | 3D T1 SG | 1.5T GE Signa | Sagittal | 124 | 0 | - | 450 | 4.2 | 10.1 | 20 |
| Florence | 3D T1 TFES | 3 T Philips Achieva | Sagittal | 191 | 1 | 1 | - | 3.7 | 8.1 | 8 |
|  | 3D T1 TFES | 1.5 T Philips | Sagittal | 175 | 1 | 1 | - | 4.6 | 25 | 30 |
| Greifswald | 3D T1 MPRAGE | 3T Verio Siemens | Sagittal | 174 | 0 | 0.9 | 900 | 2.58 | 1900 | 9 |
| IDIBAPS-HCB | 3D T1 MPRAGE | 3T Trio, Siemens | Coronal | 192 | 0 | 0.9 | 900 | 3.05 | 2000 | 9 |
| KCL_CNS | 3D T1 MPRAGE | 3T Signa HDx, GE | Coronal | 196 | 0 | 1.1 | 450 | 2.84 | 7.18 | 20 |
| KCL_CRF | 3D T1 MPRAGE | 3T MR750, GE | Sagittal | 196 | 0 | 1.3* | 400 | 3.016 | 7.312 | 11 |
| Kuopio | 3D T1 TFES | 3 T Philips Achieva | Sagittal | 190 | 0 | 1 | - | 3.7 | 8.2 | 8 |
| MNI | 3D T1 MPRAGE | 3T Trio, Siemens | Sagittal | 176 | 0 | 1 | 900 | 2.98 | 2300 | 9 |
| NYU | 3D T1 MPRAGE | 3T Allegra, Siemens | Sagittal | 128 | 1.3 | 1.3* | 1100 | 3.25 | 2530 | 7 |
| RMH | 3D T1 MPRAGE | 3T Trio, Siemens | Coronal | 192 | 0 | 0.9 | 900 | 2.21 | 1900 | 9 |
| UCSD | 3D T1 FSPGR | 3 T Discovery MR750 GE | Sagittal | 172 | 0 | 1 | 600 | 3.16 | 8.08 | 8 |
| UNAM | 3D T1 TFES | 3 T Philips Achieva | Sagittal | 186 | 0 | 1 |  | 3.69 | 8.06 | 8 |
| UNICAMP | 3D T1 TFES | 3 T Philips Achieva | Sagittal | 180 | 0 | 1 | - | 3.2 | 7 | 8 |
| UNIMORE | 3D T1 TFES | 3 T Philips Achieva | Sagittal | 170 | 0 | 1 | - | 4.6 | 9.9 | 8 |
| XMU | 3D T1 FSPGR | 3 T GE | Axial | 156 | 1.2 | 1 | 450 | 3.2 | 8.2 | 12 |

MPRAGE = magnetization prepared rapid gradient echo; FSPGR = fast spoiled gradient-recalled; SG = spoiled gradient; TFES = turbo field echo sequence; ^*^ voxel size not isometric
