# Supplementary material for: Artificial intelligence for classification of temporal lobe epilepsy with ROI-level MRI data: A worldwide ENIGMA-Epilepsy study
Source: Neuroimage Clin. 2021 Jul 24;31:102765. doi: 10.1016/j.nicl.2021.102765 (PMC8346685; doi:10.1016/j.nicl.2021.102765)
Supplement: Supplementary data 5 [file mmc5.docx]

**Supplementary Table 2.** Diffusion imaging acquisition and processing details by research site.

| Site | Scanner | Orientation | # of Slices | Voxel Size  (mm^3^) | Gradient  Directions | b-value  (mm/s^2^) | #b=0 scans | TE  (ms) | TR (ms) |
| --- | --- | --- | --- | --- | --- | --- | --- | --- | --- |
| Bonn | Siemens Trio | Axial | 160 | 1 x 1 x 1 | 60 | 1000 | 7 | 3.97 | 1300 |
| CUBRIC | GE Signa HDx | - | 60 | 2.4 mm slice thickness | 30 | 1200 | 3 | 87 | * |
| EKUT | Siemens Trio | - | 52 | 1.81 x 1.81 x 1.79 | 48 | 1200 (2x) | 6 (2x) | 93 | 9400 |
| EPICZ | GE Discovery MR750 | Axial | 80 | 2 x 2 x 2 | 27 | 1000 | 4 | 81.4 | 10000 |
| EPIGEN-Ireland | Philips Achieva | Axial | 70 | 1.75 x 1.75 x 2 | 32 | 1000 | - | 52 | 12786 |
| Florence | Philips Achieva | - | 69 | 2 x 2 x 2 | 32 | 1000 | 1 | 80 | 4000 |
| Genova | Philips Ingenia | Axial | 65 | 2 x 2 x 2 | 64 | 1000 | 1 | 90 | 7000 |
| Greifswald | Siemens Verio | - | 80 | 1.8 x 1.8 x 1.8 | 64 | 1000 | 1 | 107 | 15300 |
| Henry Ford | GE Signa | Axial | 60 | 1.96 × 1.96 × 2.6 | 25 | 1000 | 1 | 76 | 7500 |
| IDIBAPS_31DIR | Siemens Trio | Axial | 55 | 2.4 x 2.4 x 2.4 | 30 | 1000 | 1 | 90 | 6900 |
| IDIBAPS_39DIR | Siemens Trio | Axial | 64 | 1.97 x 1.97 x 2 | 36 | 1000 | 3 | 88 | 8138 |
| IDIBAPS_88DIR | Siemens Trio | Axial | 55 | 1.25 x 1.25 x 2.5 | 82 | 1000 | 6 | 98 | 7600 |
| KCL | GE Discovery MR750 | Axial | 66 | 2.4 x 2.4 x 2.4 | 32 | 1000 | 6 | 75 | * |
| Liverpool_Walton | GE Discovery MR750 | Axial | 66 | 1 x 1 x 2 | 60 | 1000 | 6 | 82 | 8000 |
| MNI | Siemens Trio | Axial | 63 | 2 x 2 x 2 | 64 | 1000 | 1 | 90 | 8400 |
| NYU | Siemens Allegra | Axial | 60 | 2.5 x 2.5 x 2.5 | 64 | 3000 | 8 | 99 | 7900 |
| Melbourne | Siemens Trio | Axial | 55 | 2.5 x 2.5 x 2.5 | 64 | 3000 | 1 | 122 | 8700 |
| UCL | GE Signa HDx | Axial | 60 | 1.875×1.875×2.4 | 52 | 1200 | 6 | 73 | * |
| UCSD | GE Discovery MR750 | Axial | 53 | 1.86 x 1.86 x 2.5 | 30 | 1000 | 2 | 82.9 | 8000 |
| UMG | Siemens Trio | - | 31 | 1.89 x 1.89 x 1.89 | 30 | 1000 | 1 | 93 | 10000 |
| UNAM | Philips Achieva | - | - | 2 x 2 x 2 | 60 | 2000 | 1 | 64.3 | 11860 |
| UNICAMP | Philips Achieva | Axial | 70 | 2 x 2 x 2 | 32 | 1000 | 1 | 61 | 8500 |
