# Supplementary material for: Artificial intelligence for classification of temporal lobe epilepsy with ROI-level MRI data: A worldwide ENIGMA-Epilepsy study
Source: Neuroimage Clin. 2021 Jul 24;31:102765. doi: 10.1016/j.nicl.2021.102765 (PMC8346685; doi:10.1016/j.nicl.2021.102765)
Supplement: Supplementary data 8 [file mmc8.docx]

**Supplementary Table 5. Performance of classification pipelines based on radial diffusivity for patients with (A) and without (B) hippocampal sclerosis**. Each section summarizes specific group comparisons (defined at the top of the section), statistical comparison of the accuracy (ACC) for support vector (SV) and deep learning (DL) pipelines, and a variety of metrics for each model, including the area under the curve (AUC), positive predictive value (PPV), negative predictive value (NPV), sensitivity (SEN), and specificity (SPC). Statistical contrasts between model performance based on real data (‘Correct Group Labels’) and shuffled data (‘Random Group Labels’) is also provided (see section 2.7).

1. **TLE-HS**

|  | **TLE-HS vs. HC** | | | | | | | | | |
| --- | --- | --- | --- | --- | --- | --- | --- | --- | --- | --- |
|  | **SV vs. DL Pipeline Classification Accuracy (ACC)** | | | | | | FDCI | | 0.7793 | |
|  | **Pipeline Performance Summary** | | | | | | | | | |
|  | **SV Pipeline** | | | | | **DL Pipeline** | | | | |
|  | **Correct Group Labels** | | **Random Group Labels** | |  | **Correct Group Labels** | | **Random Group Labels** | |  |
| **Metric** | **Mean** | **SD** | **Mean** | **SD** | **p-value** | **Mean** | **SD** | **Mean** | **SD** | **p-value** |
| **ACC** | 0.73 | 0.025 | 0.50 | 0.048 | 0.0000 | 0.71 | 0.035 | 0.50 | 0.059 | 0.0011 |
| **AUC** | 0.73 | 0.025 | 0.50 | 0.048 | 0.0000 | 0.71 | 0.035 | 0.50 | 0.059 | 0.0011 |
| **PPV** | 0.75 | 0.031 | 0.50 | 0.049 | 0.0000 | 0.73 | 0.060 | 0.49 | 0.157 | 0.0552 |
| **NPV** | 0.71 | 0.027 | 0.50 | 0.049 | 0.0000 | 0.69 | 0.045 | 0.47 | 0.151 | 0.0356 |
| **SEN** | 0.68 | 0.041 | 0.50 | 0.055 | 0.0000 | 0.65 | 0.084 | 0.50 | 0.328 | 0.3782 |
| **SPC** | 0.78 | 0.037 | 0.50 | 0.068 | 0.0000 | 0.76 | 0.075 | 0.49 | 0.337 | 0.2897 |
|  |  |  |  |  |  |  |  |  |  |  |
|  | **TLE-HS-L vs. HC** | | | | | | | | | |
|  | **SV vs. DL Pipeline Classification Accuracy (ACC)** | | | | | | FDCI | | 0.6283 | |
|  | **Pipeline Performance Summary** | | | | | | | | | |
|  | **SV Pipeline** | | | | | **DL Pipeline** | | | | |
|  | **Correct Group Labels** | | **Random Group Labels** | |  | **Correct Group Labels** | | **Random Group Labels** | |  |
| **Metric** | **Mean** | **SD** | **Mean** | **SD** | **p-value** | **Mean** | **SD** | **Mean** | **SD** | **p-value** |
| **ACC** | 0.68 | 0.035 | 0.50 | 0.055 | 0.0000 | 0.67 | 0.039 | 0.50 | 0.077 | 0.0155 |
| **AUC** | 0.68 | 0.035 | 0.50 | 0.055 | 0.0000 | 0.67 | 0.039 | 0.50 | 0.077 | 0.0155 |
| **PPV** | 0.70 | 0.040 | 0.50 | 0.055 | 0.0000 | 0.70 | 0.060 | 0.49 | 0.145 | 0.0512 |
| **NPV** | 0.67 | 0.037 | 0.50 | 0.056 | 0.0035 | 0.66 | 0.041 | 0.49 | 0.153 | 0.0762 |
| **SEN** | 0.63 | 0.060 | 0.50 | 0.067 | 0.0279 | 0.63 | 0.089 | 0.52 | 0.292 | 0.3833 |
| **SPC** | 0.73 | 0.051 | 0.49 | 0.078 | 0.0000 | 0.71 | 0.097 | 0.48 | 0.296 | 0.2643 |
|  |  |  |  |  |  |  |  |  |  |  |
|  | **TLE-HS-R vs. HC** | | | | | | | | | |
|  | **SV vs. DL Pipeline Classification Accuracy (ACC)** | | | | | | FDCI | | 0.6111 | |
|  | **Pipeline Performance Summary** | | | | | | | | | |
|  | **SV Pipeline** | | | | | **DL Pipeline** | | | | |
|  | **Correct Group Labels** | | **Random Group Labels** | |  | **Correct Group Labels** | | **Random Group Labels** | |  |
| **Metric** | **Mean** | **SD** | **Mean** | **SD** | **p-value** | **Mean** | **SD** | **Mean** | **SD** | **p-value** |
| **ACC** | 0.69 | 0.040 | 0.51 | 0.075 | 0.0011 | 0.68 | 0.055 | 0.51 | 0.072 | 0.0084 |
| **AUC** | 0.69 | 0.040 | 0.51 | 0.075 | 0.0011 | 0.68 | 0.055 | 0.51 | 0.072 | 0.0084 |
| **PPV** | 0.72 | 0.047 | 0.51 | 0.077 | 0.0011 | 0.69 | 0.096 | 0.49 | 0.173 | 0.0663 |
| **NPV** | 0.68 | 0.040 | 0.50 | 0.076 | 0.0103 | 0.68 | 0.076 | 0.50 | 0.168 | 0.0795 |
| **SEN** | 0.65 | 0.060 | 0.50 | 0.084 | 0.0506 | 0.67 | 0.128 | 0.50 | 0.315 | 0.3518 |
| **SPC** | 0.74 | 0.053 | 0.51 | 0.103 | 0.0023 | 0.70 | 0.144 | 0.51 | 0.314 | 0.3145 |
|  |  |  |  |  |  |  |  |  |  |  |
|  | **TLE-HS-L vs. TLE-HS-R** | | | | | | | | | |
|  | **SV vs. DL Pipeline Classification Accuracy (ACC)** | | | | | | FDCI | | 1.0000 | |
|  | **Pipeline Performance Summary** | | | | | | | | | |
|  | **SV Pipeline** | | | | | **DL Pipeline** | | | | |
|  | **Correct Group Labels** | | **Random Group Labels** | |  | **Correct Group Labels** | | **Random Group Labels** | |  |
| **Metric** | **Mean** | **SD** | **Mean** | **SD** | **p-value** | **Mean** | **SD** | **Mean** | **SD** | **p-value** |
| **ACC** | 0.68 | 0.041 | 0.51 | 0.058 | 0.0020 | 0.55 | 0.057 | 0.51 | 0.039 | 0.1286 |
| **AUC** | 0.68 | 0.041 | 0.51 | 0.058 | 0.0020 | 0.55 | 0.057 | 0.51 | 0.039 | 0.1364 |
| **PPV** | 0.68 | 0.044 | 0.51 | 0.059 | 0.0020 | 0.54 | 0.137 | 0.48 | 0.136 | 0.2104 |
| **NPV** | 0.68 | 0.046 | 0.51 | 0.060 | 0.0020 | 0.56 | 0.109 | 0.51 | 0.127 | 0.2026 |
| **SEN** | 0.69 | 0.063 | 0.51 | 0.094 | 0.0257 | 0.56 | 0.266 | 0.50 | 0.322 | 0.4571 |
| **SPC** | 0.67 | 0.065 | 0.52 | 0.083 | 0.0218 | 0.54 | 0.255 | 0.51 | 0.309 | 0.4714 |

1. **TLE-NS**

|  | **TLE-NS vs. HC** | | | | | | | | | |
| --- | --- | --- | --- | --- | --- | --- | --- | --- | --- | --- |
|  | **SV vs. DL Pipeline Classification Accuracy (ACC)** | | | | | | FDCI | | 0.7441 | |
|  | **Pipeline Performance Summary** | | | | | | | | | |
|  | **SV Pipeline** | | | | | **DL Pipeline** | | | | |
|  | **Correct Group Labels** | | **Random Group Labels** | |  | **Correct  Group Labels** | | **Random Group Labels** | |  |
| **Metric** | **Mean** | **SD** | **Mean** | **SD** | **p-value** | **Mean** | **SD** | **Mean** | **SD** | **p-value** |
| **ACC** | 0.62 | 0.038 | 0.51 | 0.047 | 0.0101 | 0.59 | 0.045 | 0.50 | 0.048 | 0.0313 |
| **AUC** | 0.62 | 0.038 | 0.51 | 0.047 | 0.0101 | 0.59 | 0.045 | 0.50 | 0.048 | 0.0313 |
| **PPV** | 0.62 | 0.042 | 0.51 | 0.047 | 0.0061 | 0.60 | 0.075 | 0.48 | 0.125 | 0.0758 |
| **NPV** | 0.61 | 0.038 | 0.51 | 0.048 | 0.0162 | 0.59 | 0.066 | 0.48 | 0.135 | 0.0758 |
| **SEN** | 0.60 | 0.059 | 0.51 | 0.065 | 0.0838 | 0.58 | 0.145 | 0.51 | 0.281 | 0.4121 |
| **SPC** | 0.64 | 0.057 | 0.50 | 0.067 | 0.0091 | 0.61 | 0.147 | 0.49 | 0.281 | 0.3283 |
|  |  |  |  |  |  |  |  |  |  |  |
|  | **TLE-NS-L vs. HC** | | | | | | | | | |
|  | **SV vs. DL Pipeline Classification Accuracy (ACC)** | | | | | | FDCI | | 0.0143 | |
|  | **Pipeline Performance Summary** | | | | | | | | | |
|  | **SV Pipeline** | | | | | **DL Pipeline** | | | | |
|  | **Correct Group Labels** | | **Random Group Labels** | |  | **Correct Group Labels** | | **Random Group Labels** | |  |
| **Metric** | **Mean** | **SD** | **Mean** | **SD** | **p-value** | **Mean** | **SD** | **Mean** | **SD** | **p-value** |
| **ACC** | 0.57 | 0.052 | 0.50 | 0.060 | 0.1677 | 0.54 | 0.055 | 0.50 | 0.049 | 0.1629 |
| **AUC** | 0.57 | 0.052 | 0.50 | 0.060 | 0.1677 | 0.54 | 0.055 | 0.50 | 0.049 | 0.1471 |
| **PPV** | 0.57 | 0.054 | 0.50 | 0.060 | 0.1253 | 0.52 | 0.156 | 0.45 | 0.178 | 0.2657 |
| **NPV** | 0.57 | 0.053 | 0.50 | 0.064 | 0.1576 | 0.54 | 0.137 | 0.48 | 0.169 | 0.1871 |
| **SEN** | 0.57 | 0.071 | 0.50 | 0.090 | 0.2182 | 0.57 | 0.273 | 0.48 | 0.332 | 0.4014 |
| **SPC** | 0.56 | 0.084 | 0.50 | 0.083 | 0.2071 | 0.52 | 0.274 | 0.51 | 0.328 | 0.5143 |
|  |  |  |  |  |  |  |  |  |  |  |
|  | **TLE-NS-R vs. HC** | | | | | | | | | |
|  | **SV vs. DL Pipeline Classification Accuracy (ACC)** | | | | | | FDCI | | 0.5346 | |
|  | **Pipeline Performance Summary** | | | | | | | | | |
|  | **SV Pipeline** | | | | | **DL Pipeline** | | | | |
|  | **Correct Group Labels** | | **Random Group Labels** | |  | **Correct Group Labels** | | **Random Group Labels** | |  |
| **Metric** | **Mean** | **SD** | **Mean** | **SD** | **p-value** | **Mean** | **SD** | **Mean** | **SD** | **p-value** |
| **ACC** | 0.63 | 0.059 | 0.51 | 0.083 | 0.0750 | 0.62 | 0.059 | 0.49 | 0.087 | 0.0722 |
| **AUC** | 0.63 | 0.059 | 0.51 | 0.082 | 0.0740 | 0.62 | 0.059 | 0.49 | 0.087 | 0.0794 |
| **PPV** | 0.65 | 0.073 | 0.51 | 0.087 | 0.0458 | 0.61 | 0.088 | 0.46 | 0.201 | 0.1567 |
| **NPV** | 0.61 | 0.057 | 0.51 | 0.091 | 0.1010 | 0.66 | 0.120 | 0.47 | 0.193 | 0.1268 |
| **SEN** | 0.57 | 0.095 | 0.52 | 0.121 | 0.3375 | 0.70 | 0.193 | 0.49 | 0.334 | 0.3258 |
| **SPC** | 0.68 | 0.094 | 0.50 | 0.139 | 0.0833 | 0.54 | 0.198 | 0.50 | 0.331 | 0.4588 |
|  |  |  |  |  |  |  |  |  |  |  |
|  | **TLE-NS-L vs. TLE-NS-R** | | | | | | | | | |
|  | **SV vs. DL Pipeline Classification Accuracy (ACC)** | | | | | | FDCI | | 0.5722 | |
|  | **Pipeline Performance Summary** | | | | | | | | | |
|  | **SV Pipeline** | | | | | **DL Pipeline** | | | | |
|  | **Correct Group Labels** | | **Random Group Labels** | |  | **Correct Group Labels** | | **Random Group Labels** | |  |
| **Metric** | **Mean** | **SD** | **Mean** | **SD** | **p-value** | **Mean** | **SD** | **Mean** | **SD** | **p-value** |
| **ACC** | 0.56 | 0.056 | 0.50 | 0.075 | 0.1890 | 0.55 | 0.061 | 0.50 | 0.068 | 0.1939 |
| **AUC** | 0.56 | 0.056 | 0.50 | 0.075 | 0.2209 | 0.55 | 0.060 | 0.50 | 0.068 | 0.2010 |
| **PPV** | 0.60 | 0.091 | 0.52 | 0.093 | 0.1890 | 0.54 | 0.130 | 0.47 | 0.190 | 0.2459 |
| **NPV** | 0.55 | 0.047 | 0.49 | 0.088 | 0.2418 | 0.56 | 0.146 | 0.48 | 0.191 | 0.2337 |
| **SEN** | 0.39 | 0.114 | 0.50 | 0.143 | 0.7637 | 0.59 | 0.263 | 0.52 | 0.341 | 0.4510 |
| **SPC** | 0.73 | 0.100 | 0.50 | 0.213 | 0.1835 | 0.51 | 0.265 | 0.49 | 0.339 | 0.4827 |
